# Reducing Premature Coronary Artery Disease in Malaysia by Early Identification of Familial Hypercholesterolemia Using the Familial Hypercholesterolemia Case Ascertainment Tool (FAMCAT): Protocol for a Mixed Methods Evaluation Study

**DOI:** 10.2196/47911

**Published:** 2023-06-02

**Authors:** Anis Safura Ramli, Nadeem Qureshi, Hasidah Abdul-Hamid, Aisyah Kamal, Johanes Dedi Kanchau, Nur Syahirah Shahuri, Ralph Kwame Akyea, Luisa Silva, Laura Condon, Suraya Abdul-Razak, Alyaa Al-Khateeb, Yung-An Chua, Mohamed-Syarif Mohamed-Yassin, Noorhida Baharudin, Siti Fatimah Badlishah-Sham, Aznida Firzah Abdul Aziz, Noor Alicezah Mohd Kasim, Siti Hamimah Sheikh Abdul Kadir, Joe Kai, Jo Leonardi-Bee, Hapizah Nawawi

**Affiliations:** 1 Institute of Pathology, Laboratory and Forensic Medicine (I-PPerForM) Universiti Teknologi MARA Sungai Buloh, Selangor Malaysia; 2 Department of Primary Care Medicine Faculty of Medicine Universiti Teknologi MARA Sungai Buloh, Selangor Malaysia; 3 Centre of Academic Primary Care School of Medicine, Faculty of Medicine and Health Sciences University of Nottingham Nottingham United Kingdom; 4 Cardio Vascular and Lungs Research Institute (CaVaLRI) Hospital Al-Sultan Abdullah Universiti Teknologi MARA Bandar Puncak Alam, Selangor Malaysia; 5 Department of Biochemistry and Molecular Medicine Faculty of Medicine Universiti Teknologi MARA Sungai Buloh, Selangor Malaysia; 6 Department of Family Medicine Universiti Kebangsaan Malaysia Cheras, Kuala Lumpur Malaysia; 7 Department of Pathology Faculty of Medicine Universiti Teknologi MARA Sungai Buloh, Selangor Malaysia

**Keywords:** mixed methods evaluation, study protocol, familial hypercholesterolemia, diagnostic accuracy, qualitative methods, FAMCAT, Simon Broome criteria, Dutch Lipid Clinic Criteria, genetic diagnosis, primary care, Malaysia

## Abstract

**Background:**

Familial hypercholesterolemia (FH) is predominantly caused by mutations in the 4 FH candidate genes (FHCGs), namely, *low-density lipoprotein receptor (LDLR), apolipoprotein B-100 (APOB-100), proprotein convertase subtilisin/kexin type 9 (PCSK9),* and the *LDL receptor adaptor protein 1 (LDLRAP1)*. It is characterized by elevated low-density lipoprotein cholesterol (LDL-c) levels leading to premature coronary artery disease. FH can be clinically diagnosed using established clinical criteria, namely, Simon Broome (SB) and Dutch Lipid Clinic Criteria (DLCC), and can be identified using the Familial Hypercholesterolemia Case Ascertainment Tool (FAMCAT), a primary care screening tool.

**Objective:**

This study aims to (1) compare the detection rate of genetically confirmed FH and diagnostic accuracy between the FAMCAT, SB, and DLCC in the Malaysian primary care setting; (2) identify the genetic mutation profiles, including novel variants, in individuals with suspected FH in primary care; (3) explore the experience, concern, and expectation of individuals with suspected FH who have undergone genetic testing in primary care; and (4) evaluate the clinical utility of a web-based FH Identification Tool that includes the FAMCAT, SB, and DLCC in the Malaysian primary care setting.

**Methods:**

This is a mixed methods evaluation study conducted in 11 Ministry of Health primary care clinics located at the central administrative region of Malaysia. In Work stream 1, the diagnostic accuracy study design is used to compare the detection rate and diagnostic accuracy of the FAMCAT, SB, and DLCC against molecular diagnosis as the gold standard. In Work stream 2, the targeted next-generation sequencing of the 4 FHCGs is used to identify the genetic mutation profiles among individuals with suspected FH. In Work stream 3a, a qualitative semistructured interview methodology is used to explore the experience, concern, and expectation of individuals with suspected FH who have undergone genetic testing. Lastly, in Work stream 3b, a qualitative real-time observation of primary care physicians using the “think-aloud” methodology is applied to evaluate the clinical utility of a web-based FH Identification Tool.

**Results:**

The recruitment for Work stream 1, and blood sampling and genetic analysis for Work stream 2 were completed in February 2023. Data collection for Work stream 3 was completed in March 2023. Data analysis for Work streams 1, 2, 3a, and 3b is projected to be completed by June 2023, with the results of this study anticipated to be published by December 2023.

**Conclusions:**

This study will provide evidence on which clinical diagnostic criterion is the best to detect FH in the Malaysian primary care setting. The full spectrum of genetic mutations in the FHCGs including novel pathogenic variants will be identified. Patients’ perspectives while undergoing genetic testing and the primary care physicians experience in utilizing the web-based tool will be established. These findings will have tremendous impact on the management of patients with FH in primary care and subsequently reduce their risk of premature coronary artery disease.

**International Registered Report Identifier (IRRID):**

DERR1-10.2196/47911

## Introduction

Familial hypercholesterolemia (FH) is an autosomal dominant genetic disorder characterized by elevated low-density lipoprotein cholesterol (LDL-c) that leads to premature atherosclerotic cardiovascular disease, particularly coronary artery disease (CAD) [1,2]. Genetic mutations in 3 FH candidate genes, namely, *low-density lipoprotein receptor* (*LDLR*), *apolipoprotein B-100* (*APOB-100*)*,* and *proprotein convertase subtilisin/kexin type 9* (*PCSK9*), are responsible for the autosomal dominant form of FH [2], while other rare mutations in the *LDL receptor adaptor protein 1* (*LDLRAP1*) gene and *apolipoprotein E* (*APOE*) gene have been reported to be responsible for the recessive form of FH [3,4]. Mutations in the *ATP-binding cassette subfamily G members 5* and *8* (*ABCG5 and ABCG8*, respectively) genes have been associated with sitosterolemia, an autosomal recessive disorder characterized by increased plant sterol levels [5].

Phenotypically, FH presents in the form of either heterozygous FH (HeFH) or homozygous FH (HoFH) [1,2]. An estimated 70%-90% of cases are HeFH, while HoFH is less common [6,7]. Individuals with HeFH typically present with LDL-c levels of more than 4.9 mmol/L, whereas those with HoFH present with LDL-c levels of more than 13 mmol/L [6,7]. If left untreated, men with FH have a 50% risk of developing CAD by the age of 50, and women with FH have a 30% risk of developing CAD by the age of 60 [8]; however, most individuals with HoFH experience severe CAD before the age of 20 and generally do not survive beyond 30 years of age [6]. Early detection of FH and treatment with lipid-lowering therapies can effectively prevent premature CAD by extending CAD-free life up to 18 years, compared with untreated individuals [9].

Globally, the prevalence of HeFH ranges from 1 in 200 to 1 in 500 in various populations [10], while the prevalence of HoFH is estimated to range from 1 in 160,000 to 1 in 300,000 [6]. In Malaysia, a recent study [11] has estimated that the prevalence of clinically diagnosed FH was 1 in 100. Based on the current total population of 32 million people, a staggering 320,000 individuals are estimated to have FH [11]. A majority of these cases were previously undiagnosed and lipid-lowering medications were prescribed to 30.5%-54.5% of these individuals, but none of them achieved the therapeutic LDL-c target [11]. The under-detection and under-treatment of FH in the Malaysian population resulted in lost opportunities to prevent premature CAD [11]. This common autosomal dominant condition has undeniably contributed to the high prevalence of premature CAD which accounted for 10%-15% of acute coronary syndrome in Malaysia [12]. According to a national report, 23.8% of patients admitted with acute coronary syndrome were younger than 50 years old [13]. Among patients with confirmed premature CAD, 45.5% had clinically diagnosed FH [14]. Therefore, identifying and treating FH early, and subsequently reducing the incidence of premature CAD is a key national priority in Malaysia [15].

Improving FH identification in primary care has been recognized as a cost-effective strategy to reduce premature CAD [16]. In Malaysia, primary care physicians (PCPs) are ideally positioned at the forefront of primary care service where they manage common atherosclerotic cardiovascular disease risk factors such as hypercholesterolemia, hypertension, obesity, and diabetes [17]. However, substantial gaps in knowledge regarding FH have been identified, including knowledge on prevalence, inheritance, and risk of CAD as well as on awareness of FH clinical guidelines and diagnostic criteria [18]. These gaps in knowledge, awareness, and practice regarding FH among PCP could be a significant contributing factor to the poor detection of FH in Malaysia [18]. Efforts are currently being undertaken to address these gaps through a partnership with the FH Ten Countries Study to improve awareness of FH among PCP in Malaysia [10,18].

Currently, individuals with FH tend to be discovered incidentally in primary care practice. It has been suggested that primary care should be the key target area to increase identification of new FH index cases and to initiate cascade screening [19-22]. Researchers in the United Kingdom have shown that early identification of FH is achievable in primary care [23-26]. However, the 2 established clinical criteria, namely, Simon Broome (SB) and Dutch Lipid Clinic Criteria (DLCC), are of limited use for case-finding in primary care [24]. These clinical diagnostic criteria were originally developed from secondary care FH registries. Furthermore, these criteria would require a comprehensive physical examination (tendon xanthoma and arcus cornealis), recording of family history, and genetic mutation testing, which are not routinely assessed or inadequately documented in primary care. As a consequence, researchers in the United Kingdom have developed a new tool, the Familial Hypercholesterolemia Case Ascertainment Tool (FAMCAT), based on a risk prediction algorithm developed and validated using primary care databases [26].

The FAMCAT has been shown to perform well as an initial case ascertainment tool to identify FH in the UK primary care setting when it was tested among 3.7 million British patients with cholesterol measurements in primary care electronic health records [25]. Compared with other established diagnostic criteria, the FAMCAT is 20% more accurate in identifying FH than SB criteria and 15% more accurate than DLCC [25,26]. Targeted case-finding for FH in the primary care setting has already been shown to be cost-effective by prioritizing those with the highest likelihood of having FH for further assessment and genetic testing in secondary care [27-29]. This measure would efficiently allocate resources toward those who would benefit the most [27,29].

The performance of the FAMCAT in the United Kingdom has been well recognized [23-26]. However, its performance in the Malaysian primary care setting remains unknown. To improve identification of patients with FH in the Malaysian primary care, we developed a web-based FH Identification Tool to collect key information to identify individuals with suspected FH using the FAMCAT, DLCC, and SB criteria. Using these criteria, patients were screened for features of FH. Those who were suspected to have FH clinically were offered genetic testing. Genetic testing is not routinely available in Malaysia. However, it can be offered if resources are available as it is the gold standard to diagnose FH [30]. We hypothesize that the rate of detection of genetic mutations using the FAMCAT will be higher compared with the SB criteria and DLCC score, thus showing better performance.

Overall, this study aims to enhance detection of individuals with high probability of FH in the Malaysian primary care setting who can then be genetically tested and subsequently referred for further clinical management and cascade screening of family members in multidisciplinary specialized lipid clinics, maximizing efficient use of limited resources. The specific objectives of this study are to (1) compare the detection rate of genetically confirmed FH and the diagnostic accuracy between the FAMCAT and the established clinical diagnostic criteria (SB and DLCC) in the Malaysian primary care setting; (2) identify the genetic mutation profiles, including novel variants, in individuals with suspected FH in the Malaysian primary care setting; (3) explore the experience, concern, and expectation of individuals with suspected FH who have undergone genetic testing in the Malaysian primary care setting; and (4) evaluate the clinical utility of a web-based FH Identification Tool that includes the FAMCAT, SB, and DLCC in the Malaysian primary care setting.

## Methods

### Study Overview

This is a mixed methods evaluation study that used both the quantitative and qualitative research methods to answer complex research questions, and to understand the process and to strengthen the impact evaluation [31]. Methodologically, this study was divided into Work streams 1, 2, and 3. In Work stream 1, the diagnostic test accuracy study design is used to compare the detection rate of genetically confirmed FH and discriminatory accuracy between the FAMCAT, SB, and DLCC in the Malaysian primary care setting. In Work stream 2, the targeted next-generation sequencing (NGS) is used to identify the genetic mutation profiles among individuals with suspected FH in the Malaysian primary care setting. In Work stream 3a, a qualitative semistructured interview methodology is used to explore the experience, concern, and expectation of individuals with suspected FH who have undergone genetic testing in the Malaysian primary care setting. Lastly, in Work stream 3b, a qualitative real-time observation of PCPs using the “think-aloud” methodology is applied to evaluate the clinical utility of a web-based FH Identification Tool containing the FAMCAT, SB, and DLCC in the Malaysian primary care setting.

### Work Stream 1: Comparing the Detection Rate and Diagnostic Accuracy of Genetically Confirmed FH in the Malaysian Primary Care

#### Study Design and Setting

This is a diagnostic test accuracy study. The study was conducted at 11 Ministry of Health (MOH) primary care clinics located in the urban and suburban areas at the central administrative region of Malaysia (in the states of Selangor, Kuala Lumpur, and Putrajaya). These areas were chosen due to their commutable proximity to the Universiti Teknologi MARA (UiTM) Lipid Specialist Clinic where patients who are genetically confirmed to have FH are referred for further management and cascade screening of their family members.

#### Site Selection

To be eligible for selection, the clinics must be equipped with an electronic medical record (EMR) system, have minimum attendance of 500 patients per day, and be led by a family medicine specialist (FMS).

In total, there were 21 MOH primary care clinics (15 clinics in Selangor and 6 clinics in Kuala Lumpur and Putrajaya) that fulfilled the aforesaid selection criteria. The FMS leading these 21 clinics were invited for a briefing to introduce the study. Out of the 21 clinics, 11 clinics were interested in participating and therefore were included in the study (6 are located in Selangor and the other 5 are located in Kuala Lumpur and Putrajaya).

The site selection for this study was completed in February 2020. Table 1 shows the demographic characteristics of the study sites. All the clinics are located in the urban and suburban areas. The population served ranged from approximately 78,000 to 500,000, with most from the low to middle household income groups. All the clinics have multidisciplinary team members consisting of doctors (FMS and medical officers), assistant medical officers, nurses, dietitians/nutritionists, and pharmacists.

**Table 1:** Demographic characteristics of the study sites.

Number	Clinic name	Area	Total number of populations served, n	Description of populations served	Total number of multidisciplinary team members, n
1	Precinct 18, Putrajaya	Suburban	91,900	Middle to high income Majority are civil servants	180
2	Section 7, Shah Alam	Urban	511,153	Low to middle income Majority are working in the private sector	103
3	Sungai Buloh	Suburban	466,163	Low to middle income Majority are unemployed and self-employed	183
4	Jinjang	Urban	132,272	Low to middle income Majority are self-employed and unemployed	205
5	Pandamaran	Urban	150,000	Low to middle income A mixture of private sector workers and civil servants	200
6	Kelana Jaya	Suburban	180,000	Low to middle income A mixture of private sector workers and civil servants	171
7	Taman Ehsan	Suburban	78,693	Low to middle income A mixture of private sector workers and civil servants	103
8	Selayang Baru	Urban	212,164	Low to middle income A mixture of private sector workers and civil servants	150
9	Tanglin	Urban	400,000	Low to middle income A mixture of private sector workers and civil servants	170
10	Kuala Lumpur	Urban	105,000	Middle income A mixture of private sector workers and civil servants	217
11	Batu Muda	Suburban	180,000	Low to middle income A mixture of private sector workers and civil servants	158

#### Study Population

Patients registered at the 11 MOH primary care clinics were recruited according to the inclusion and exclusion criteria. This study included patients aged 18 years or older and who had an LDL-c level of 4.0 mmol/L or more recorded in their EMR. Those who were previously diagnosed with FH by genetic testing, were pregnant, or did not have the mental capacity to provide informed consent were excluded.

#### Study Tool and Variables

**Figure 1:**
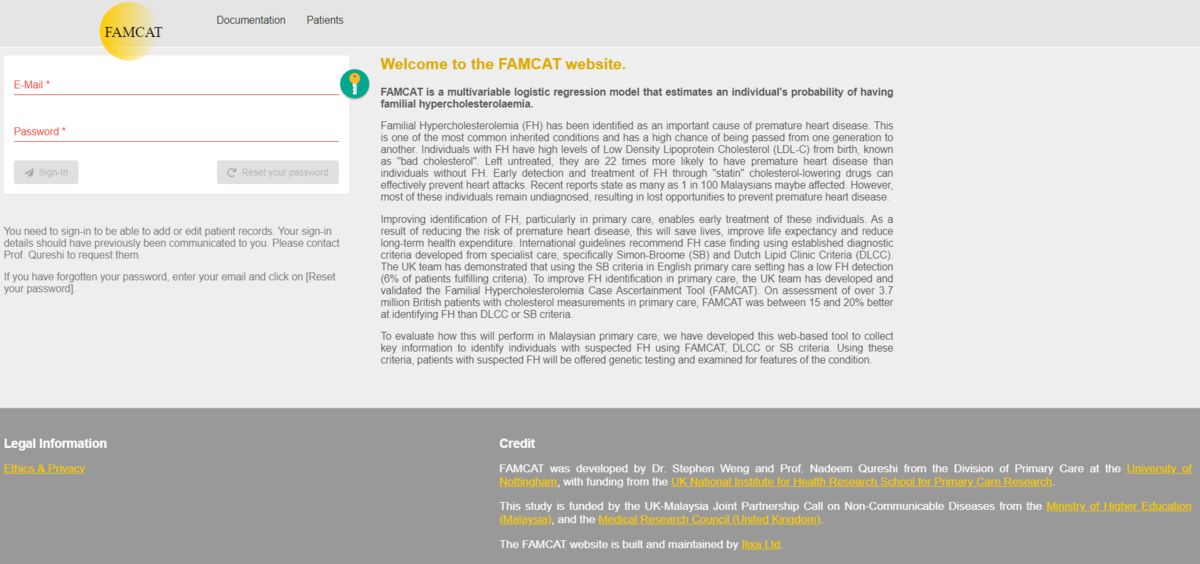
Interface of the FAMCAT web-based FH Identification Tool.

This study used the FAMCAT web-based FH Identification Tool, developed by and based at the University of Nottingham, UK. This web-based tool contains 3 index tests, namely, the FAMCAT, SB, and DLCC. Figure 1 shows the interface of the FAMCAT web-based FH Identification Tool. Table 2 shows the variables needed to identify patients with suspected FH using the FAMCAT web-based FH Identification Tool.

The variables entered into the FAMCAT web-based FH Identification Tool identified patients with suspected FH who were indicated to have genetic testing based on either one of the following definitions:

- The FAMCAT algorithm relative risk score >1 (high probability of FH).
- Clinical diagnosis of “definite” or “possible” FH by SB criteria.
- DLCC score ≥6 (clinical diagnosis of “definite” or “probable” FH by DLCC).

The disease of interest is the HeFH and the reference standard for diagnosis is genetically confirmed HeFH determined by targeted NGS of pathogenic variants (PVs) in 4 FH candidate genes (ie, *LDLR, APOB-100, PCSK9*, and *LDLRAP1*).

Full details of the genetic testing methods are presented in Work stream 2. The laboratory staff analyzing the samples and the specialist interpreting the genetic test results were blinded to the index tests results.

**Table 2:** Clinical diagnostic variables for the FAMCAT^a^, SB^b^, and DLCC^c^.

Index tests	SB criteria	DLCC	FAMCAT
Lipid levels	Total cholesterol >7.5 mmol/L LDL-c^d^ >4.9 mmol/L	LDL-c ≥4 mmol/L	The highest LDL-c ever measured The highest total cholesterol ever measured Triglycerides during LDL-c measurement Age during LDL-c measurement Lipid-lowering treatment during LDL-c measurement
Physical examination	Tendon xanthomas	Tendon xanthomas Corneal arcus <45 years of age	Nil
Personal history	Nil	Personal history of premature CAD^e^, cerebrovascular accident, or peripheral vascular disease (<55 years in men, <60 years in women)	Personal history of premature myocardial infarction (<55 years in men, <60 years in women) Diagnosis of diabetes Diagnosis of chronic kidney disease
Family history	Tendon xanthomas in a first- or second-degree relative Family history of premature myocardial infarction <60 years in a first-degree relative or <50 years in a second-degree relative Family history of elevated total cholesterol >7.5 mmol/L in a first- or second-degree relative Family history of elevated total cholesterol >6.7mmol/L in a child or siblings ≤16 years old	A first-degree relative with tendon xanthomas or corneal arcus A first-degree relative with premature CAD, cerebrovascular accident, or peripheral vascular disease (<55 years in men, <60 years in women) A first-degree relative with LDL-c >95th percentile Children <18 years with LDL-c >95th percentile	Family history of premature myocardial infarction (<55 years in men, <60 years in women) Family history of familial hypercholesterolemia

^a^FAMCAT: Familial Hypercholesterolemia Case Ascertainment Tool.

^b^SB: Simon Broome.

^c^DLCC: Dutch Lipid Clinic Criteria.

^d^LDL-c: low-density lipoprotein cholesterol.

^e^CAD: coronary artery disease.

#### Patients Recruitment and Sampling Method

Patient recruitment was conducted by the research assistants (RAs) under the supervision of clinician investigators, who were the FMS. A list of patients with LDL-c levels of 4.0 mmol/L or more was extracted from the EMR. These patients were then stratified into 4 groups (≥7.0 mmol/L, 6.0-6.9 mmol/L, 5.0-5.9 mmol/L, and 4.0-4.9 mmol/L). Patients in these 4 groups were invited to participate via social media messaging service. This comprised a concise invitation message and a flyer containing information about the study. Invitations were sent to all patients in the highest LDL-c groups of 7.0 mmol/L or more and 6.0-6.9 mmol/L. Equal numbers of patients in the other 2 groups (5.0-5.9 mmol/L and 4.0-4.9 mmol/L) were invited to participate. If the patient did not respond to the invitation message sent via the social media messaging service, the RA followed up with a telephone call. Those who agreed to participate were given an appointment to be screened according to the inclusion and exclusion criteria at the primary care clinic.

At the primary care clinic, the patients received the study information sheet and informed consent form. This is available in either Malay or English language according to patients’ preference. It contains important information pertaining to the study and included background; purpose; benefit and risk; information regarding participation, including possibility of genetic testing (if indicated), study procedure, confidentiality status, and contact information. The RA gave the information verbally when the patients required further information or clarification.

Those who verbally consented to participate were screened for eligibility according to the inclusion and exclusion criteria. Screening for the eligibility criteria was conducted by the RA through structured interviewing of the patients. Those who fulfilled the eligibility criteria and agreed to participate were recruited; and written informed consent was obtained by the RA.

Patients were recruited until the sample size was achieved. Sample size calculation for this study is described later in this manuscript.

#### Data and Sample Collection Procedures

Data collection at the primary care clinics was conducted by the RA under the supervision of the clinician investigators. Prior to data collection, a training workshop for the RA and the clinician investigators was conducted to ensure standardization of study procedures. The training included physical examinations to identify tendon xanthomata and corneal arcus, and taught the participants how to use the FAMCAT web-based FH Identification Tool. At the primary care clinic, the RA also had an on-site hands-on training on how to interview patients, review the EMR, identify clinical signs of FH, and enter data into the FAMCAT web-based FH Identification Tool. During the first 2 months of the study, the clinician investigator directly observed the RA during the data collection and physical examination procedures. Further, they were trained on how to counsel patients for genetic testing.

Data were collected using the data collection form and standardized questionnaires, both of which have been translated into the Malay language. Participants were requested to fill in the sociodemographic data (age, ethnicity, education level, marital status, household income) and the Family History Questionnaire [32]. Clinical data (ie, significant medical history, medication history, and previous lipid profile results [total cholesterol, triglycerides, and LDL-c]) were retrieved from the EMR in the presence of the patients. Physical examination to identify tendon xanthomata and corneal arcus was performed by the RA, supervised by the clinician investigators. To identify patients who may need immediate medical attention, the WHO Rose Angina Questionnaire [33] and Edinburgh Claudication Questionnaire [34] were administered.

All of the variables that were collected on paper were double entered by the RA into a Microsoft Excel spreadsheet and the FAMCAT web-based FH Identification Tool to identify patients with suspected FH who were indicated for genetic testing. The criteria for genetic testing have previously been described in the “Study Tool and Variables” section.

Patients with suspected FH who were indicated to have genetic testing were given another set of patient information sheet and informed consent form in either Malay or English language. It contains important information pertaining to genetic testing. Participants were counseled regarding their risk of having FH, the pattern of inheritance, the genetic testing procedure, confirmation of diagnosis after the genetic testing, further management, and the need for cascade screening of family members once they are genetically diagnosed. Written informed consent was obtained from those who agreed to participate. Approximately 4 mL of venous blood sample was collected from each participant into an ethylenediaminetetraacetic acid (EDTA) tube. These samples were kept in a temperature-controlled container and were delivered to the Institute of Pathology, Laboratory and Forensic Medicine (I-PPerForM) Genetic Laboratory within 3 hours.

#### Sample Size Calculation

A previous study, in a UK population, found the detection rate for genetically confirmed FH using the FAMCAT to be 28% [35]. Therefore, a sample size of 310 will allow for a single proportion of 28% to be estimated with a 95% confidence level within a 5% margin (ie, from 23% to 33%).

#### Study Outcomes and Process Measures

##### Primary Outcomes

- Comparison of detection rate of genetically confirmed FH between the FAMCAT, SB, and DLCC.

##### Secondary Outcomes

- Sensitivity and specificity;
- Area under the curve;
- Positive and negative predictive values;
- Diagnostic odd ratio.

##### Process Measures

- Number of patients who are screened through the EMR and invited to participate.
- Number and proportion of patients who disagree to participate or do not respond (out of those invited).
- Number and proportion of patients who agree to participate and are given appointment to be seen at the primary care clinic (out of those invited).
- Number and proportion of patients who withdraw after being given an appointment (out of those given an appointment).
- Number and proportion of patients who are assessed for eligibility according to the inclusion/exclusion criteria at the primary care clinic (out of those given an appointment).
- Number and proportion of patients who are excluded due to noneligibility (out of those who are assessed at the clinic).
- Number and proportion of patients who are eligible, recruited, and for whom written informed consent was obtained (out of those who are assessed at the clinic).
- Number and proportion of patients with complete diagnostic variables data entered into the web-based FH Identification Tool (out of those who are eligible and recruited).
- Number and proportion of patients who fulfilled the genetic testing criteria (out of those who are eligible and recruited).
- Number and proportion of patients who give consent and have blood sample taken for genetic analysis (out of those who fulfilled the genetic testing criteria).
- Number and proportion of patients who are identified to have PV in any of the 4 FH candidate genes (out of those who have blood sample taken for genetic analysis).
- Number and proportion of patients who agree to be referred to the lipid specialist for further care (out of those who have PV in any of the 4 FH candidate genes).

**Figure 2:**
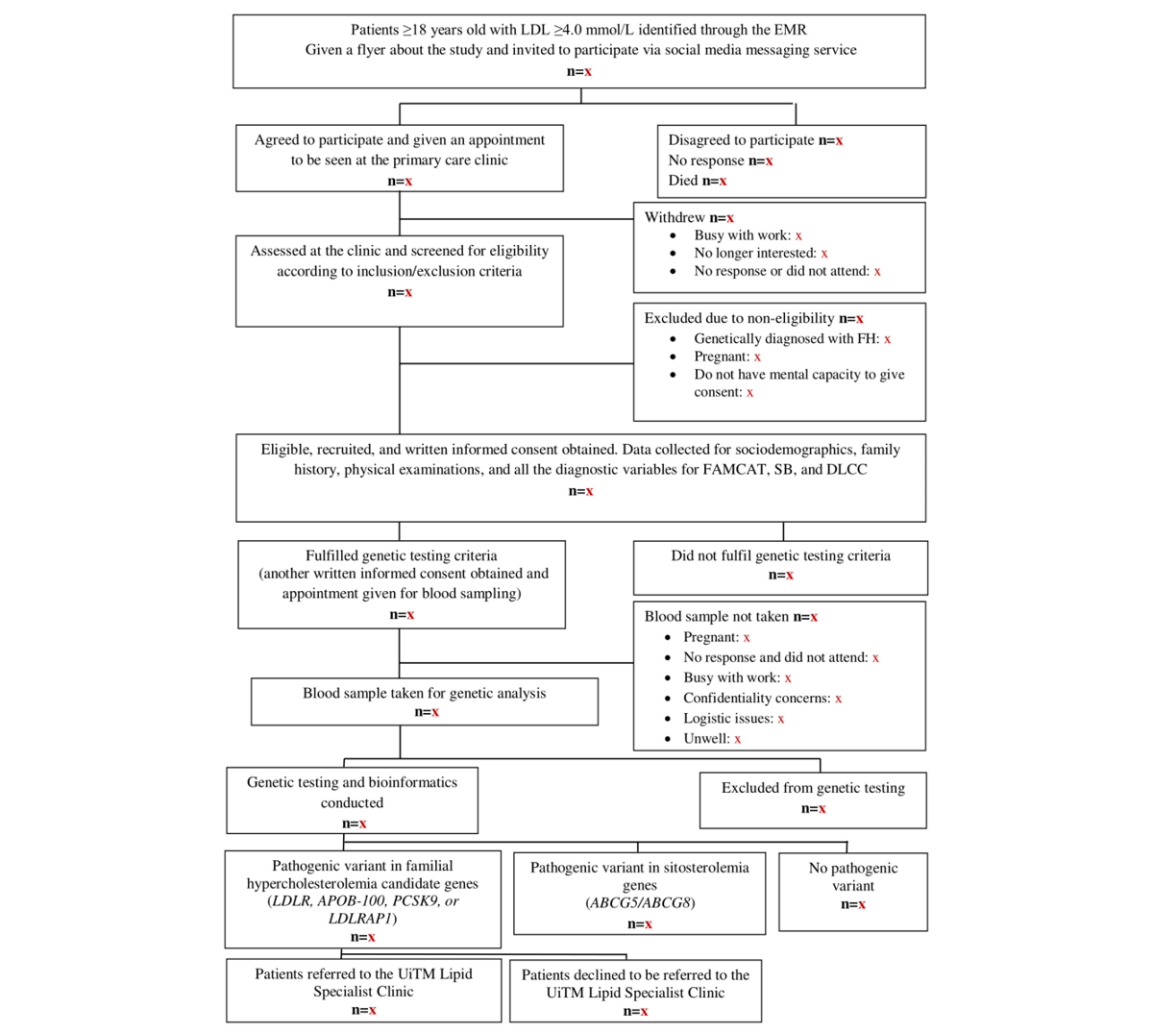
Flowchart of the participants screening and recruitment.

Figure 2 shows the flowchart of participants screening and recruitment at the primary care clinics.

#### Statistical Analysis Plan

The demographics and clinical characteristics of the participants included in the study will be reported using descriptive measures, where frequencies (with percentages) will be used for categorical outcomes and either means (with SDs) or medians (with IQRs) will be used for continuous outcomes. Measures of diagnostic accuracy (detection rate, sensitivity, specificity, positive and negative predictive values, area under the curve, and diagnostic odds ratio) for each of the 3 index tests will be reported with their corresponding 95% CIs, with genetic testing as the reference standard. The diagnostic accuracy of the FAMCAT compared with SB and DLCC will be conducted using paired analyses by constructing a joint classification table and estimating the relative diagnostic odds ratio and relative likelihood ratios, with corresponding 95% CIs. Stratified analyses will be conducted to assess the impact of ethnicity (genotyping profile) and sensitivity analyses will be conducted excluding people with hypothyroidism. Process outcome measures will be reported as frequencies (with percentages).

### Work Stream 2: Identifying Genetic Mutation Profiles of Individuals With Suspected FH

#### Study Design and Setting

Targeted NGS of the 4 FH candidate genes (FHCGs), namely, *LDLR, APOB-100, PCSK9,* and *LDLRAP1,* and 2 hypercholesterolemia-associated genes (HCAGs), namely, *ABCG5* and *ABCG8.* The study is performed at the I-PPerForM Genetic Laboratory located in UiTM Sungai Buloh Campus, Selangor, Malaysia.

#### Sample Collection

The EDTA tubes containing venous blood samples were delivered in a temperature-controlled container from the primary care clinics to the I-PPerForM Genetic Laboratory within 3 hours. Once received, the whole blood was stored in a freezer at –20°C before further analysis.

#### DNA Extraction

The DNA extraction was conducted by an RA who was blinded to the index tests results (FAMCAT, SB, and DLCC) using standardized method [36]. The frozen blood samples were thawed at room temperature before being subjected to DNA extraction using MasterPure DNA Purification Kit for Blood Version II (Lucigen Corporation). A total of 200 µL whole blood and red cell lysis solution (600 µL) were pipetted and mixed in a microcentrifuge tube. The mixture was incubated at room temperature for 5 minutes and then briefly vortexed. After centrifuging for 25 seconds, the supernatant was removed, leaving approximately 25 µL of pellet (white blood cells). The white blood cells were resuspended in 300 µL tissue and cell lysis solution by pipetting. RNase A (1 µL) was added and thoroughly mixed. The samples were then incubated for 30 minutes at 37°C. The samples were placed on ice for 3-5 minutes, followed by addition of the MasterPure Complete protein precipitation reagent (175 µL) into the lysed sample. The mixture was vigorously vortexed for 10 seconds. The debris was pelleted by centrifugation for 10 minutes at 10,000*g* or more in a microcentrifuge. The supernatant was transferred to a clean centrifuge tube and the pellet was discarded. Isopropanol (500 µL) was added to the recovered supernatant. The DNA was pelleted by centrifugation at 4°C for 10 minutes in a microcentrifuge. The isopropanol was carefully removed, without dislodging the DNA pellet. The DNA pellet was rinsed 2 times with 70% ethanol (EtOH; Fisher Scientific), without dislodging the pellet. All the residual EtOH was removed. Approximately 3-9 μg of DNA was acquired, which was then resuspended in 35 µL of Tris-EDTA (TE) buffer (Sigma). The DNA was then quantitated. The extracted DNA was stored in a –20°C freezer to preserve its integrity.

#### Quantification and Quality Control

##### QuickDrop Quantification

The concentration and purity of samples were determined using the SpectraMax QuickDrop Micro-Volume Spectrophotometer (Molecular Devices). TE buffer (1 µL) was then used as the blank and 1 µL sample was pipetted onto the sample port. The reading for the concentration (ng/µL) and purity were recorded. Samples with concentration of 20 ng/µL or more and purity of A260/A280=1.70-2.00 were subjected to electrophoresis that was performed to clarify the intactness of DNA.

##### Qubit Quantification

The Qubit dsDNA HS (High Sensitivity) Assay Kit (Thermo Fisher Scientific) was used to further assess DNA quality. Samples with concentration of 50 ng/µL or more based on the QuickDrop readings were diluted to 18-30 ng/µL in case the reading was underestimated before proceeding to Qubit quantification.

The Qubit working solution was prepared by diluting the Qubit dsDNA HS Reagent in the Qubit dsDNA HS Buffer with a ratio of 1:200. The Qubit dsDNA HS assay requires 2 standards for calibration. The standards were prepared by mixing 190 μL Qubit working solution and 10 μL of each Qubit standard. The solution was then mixed by vortexing for 2-3 seconds. For sample preparation, 2 μL of the sample was added to 198 μL of the Qubit working solution. The solution was then mixed by vortexing for 2-3 seconds. The tubes were allowed to incubate at room temperature for 2 minutes before being read by the fluorometer. The actual concentration of DNA was obtained by calculating the readings from Qubit and times with the number of dilutions.

##### Ethanol Purification

One part of sodium acetate solution (3.0 M; pH 5.2; Sigma) and 2 parts of cold absolute EtOH were added to 10 parts of the DNA sample volume. The mixture was vortexed for 10 seconds and kept in a –70°C freezer for 20 minutes. The mixture was then transferred into a column tube and centrifuged for 10 minutes, at 14,000*g* in 4°C. The supernatant was carefully discarded without moving the DNA pellet, which was later washed with 500 µL of 70% EtOH, followed by centrifugation for another 10 minutes, at 14,000*g* in 4°C. The supernatant was discarded and the washed pellets were air dried. The pellets were resuspended in 40 µL elution buffer (EB) buffer and requantified using QuickDrop, followed by agarose gel electrophoresis and Qubit fluorometer reading.

#### Next-Generation Sequencing

##### Custom Targeted Gene Sequencing

The NGS [37] was run using an AmpliSeq Custom DNA panel (Illumina), which was designed using the Illumina DesignStudio Sequencing Assay Designer. The targeted NGS employed the amplicon sequencing method, where PCR primers were used to amplify the sequences of interest. Samples used for amplicon sequencing were transformed into libraries and enriched via PCR amplification individually and barcoded by ligating the indexes into the amplicons. The libraries were then pooled and analyzed using a bench-top sequencer. The panel in this study was designed to identify the 4 FHCGs, namely, *LDLR, APOB-100, PCSK9,* and *LDLRAP1,* and the HCAGs, namely, *ABCG5* and *ABCG8,* with an overall gene coverage of 98.61% [38-40].

##### Quantification and Dilution of DNA

The samples were diluted to an intermediate concentration of about 20-50 ng/µL using low TE followed by requantification using the Qubit fluorometer. The samples were then further diluted into the desired final concentration of 3.5 ng/µL using low TE.

##### Amplification of DNA Targets

For each sample, 7 µL of 5× AmpliSeq HiFi Mix and 10.5 µL DNA samples (3.5 ng/µL) were combined in a fresh 1.5-mL microcentrifuge tube to bring the total volume of the master mix to 17.5 µL. About 5 µL of the master mix was transferred into a 96-well PCR plate. Because of the complexity of the panel design, each sample was separated into 3 different wells and separately added with different 5 µL of 2× AmpliSeq Custom DNA Panel Pool (the primer pool). In total, each well contained 5 µL sample master mix and 5 µL primer pool for a total of 10 µL per well. The plate was properly sealed and briefly centrifuged. The plate was placed in a preprogrammed thermal cycler and run with the following settings: preheat lid option, 105°C; reaction volume, 10 µL; 99°C, 2 minutes; 19 cycles of –99°C for 15 minutes and –60°C for 4 minutes; and 10°C for up to 24 hours.

##### Partial Digestion of Amplicons

After PCR, the 3 separated products amplified from the same sample were then combined into 1 well, making the total volume per sample as 30 µL. Then, 3 µL of the FuPa reagent was added to each combined sample. The plate was sealed, briefly vortexed at 1600 rpm for 1 minute, followed by centrifugation at 280*g* for 1 minute. The plate was placed in the thermal cycler and run with the following settings: preheat lid, 105°C; reaction volume, 33 µL; 50°C for 10 minutes; 55°C for 10 minutes; 62°C for 10 minutes; and 10°C for up to 1 hour.

##### Ligate Indexes

Switch solution (6 µL), AmpliSeq CD indexes (3 µL), and finally DNA ligase (3 µL) were added sequentially into each well containing the digested amplicons (33 µL) to a total volume of 45 µL for each sample. The plate was then sealed, vortexed, centrifuged, and placed into the thermal cycler and run with the following settings: preheat lid, 105°C; reaction volume, 45 µL; 22°C for 30 minutes; 68°C for 5 minutes; 72°C for 5 minutes; and 10°C for up to 24 hours.

##### Cleanup Library

A total of 45 µL of AMPure XP beads were added to each library. The plate was then sealed, vortexed, centrifuged, and incubated at room temperature for 5 minutes. The plate was then placed on a magnetic stand until the mixture became clear (approximately 2 minutes). While still on the magnetic stand, the plate was unsealed, and the supernatant was carefully pipetted out from each well. The beads were washed by adding 150 µL freshly prepared 70% EtOH to each well, followed by incubation at room temperature until the solution became clear (approximately 30 seconds). Without disturbing the pellets, the supernatant was again discarded from each well. The washing step was repeated once again. The plate was sealed, briefly centrifuged, and placed on the magnetic stand in the same position as before. The plate was unsealed and all residual EtOH was removed from each well.

##### Amplify Library

In a fresh 1.5 μL microcentrifuge tube, 1125 µL of 1× Lib Amp Mix was combined with 125 µL of 10× Library Amp Primers for a total of 1250 µL amplification master mix. The master mix was vortexed and centrifuged briefly. The plate was removed from the magnetic stand and 50 µL of the master mix was added to each well and the plate was sealed again, vortexed, centrifuged, and placed into the thermal cycler and run with the following settings: preheat lid, 105°C; reaction volume, 50 µL; 98°C for 2 minutes; 7 cycles of 98°C for 15 seconds and 64°C for 1 minute; and 10°C for up to 24 hours.

##### Second Cleanup

About 25 µL of AMPure XP beads were added to each well containing approximately 50 µL library and the plate was sealed again. The library was vortexed, centrifuged briefly, and incubated at room temperature for 5 minutes. The plate was placed on the magnetic stand for another 5 minutes until the liquid is clear and the plate was unsealed. The entire supernatant that contains the desired amplicon library (approximately 75 µL) was transferred to a new plate and 60 µL of AMPure XP beads were added to each well in the new plate. The plate was briefly vortexed, centrifuged, and incubated at room temperature. After 5 minutes, the plate was placed on the magnetic stand for another 5 minutes until the liquid cleared. The plate was unsealed. Without disturbing the beads, the supernatant was discarded from each well. The beads were washed by adding 150 µL freshly prepared 70% EtOH to each well, followed by incubation at room temperature until the solution became clear (approximately 30 seconds). Without disturbing the pellets, the supernatant was discarded from each well. The washing step was repeated once again. All residual EtOH was pipetted out, and the plate was air dried on the magnetic stand for 5 minutes. Low TE (30 µL) was added to each well and mixed well. The plate was sealed again, vortexed, and centrifuged briefly to disperse the beads. It was then placed on the magnetic stand until the liquid cleared and then unsealed. About 27 μL supernatant that contains the amplicon library was transferred to a new plate.

##### Libraries Checking

Each library was subjected to Qubit quantification to ensure the concentrations were approximately 1-10 ng/µL.

##### Bioanalyzer Reading

Each sample was diluted to 0.5-1.0 ng/µL before the bioanalyzer step. Size distribution of the library was checked using the Agilent High Sensitivity DNA Kit (Agilent Technologies). Nearly 15 mL of high-sensitivity DNA dye concentrate was added to a vial containing high-sensitivity DNA gel matrix. The mixture was vortexed and transferred to a spin filter, and subsequently centrifuged to obtain the elution. The gel-dye mix elution was allowed to equilibrate to room temperature for 30 minutes before use. A high-sensitivity DNA chip was put on the chip priming station. About 9 μL of the gel-dye mix was pipetted into the loading well on the chip. A priming plunger was used to push the gel-dye mix to the entire sample wells on the chip. About 5 μL marker was pipetted into each sample well and a ladder well. Nearly 1 μL high-sensitivity DNA ladder was also pipetted in the ladder well. Then, 1 μL sample was also pipetted into each sample well. The chip was vortexed for 1 minute at 2400 rpm, and then run in the Agilent 2100 Bioanalyzer instrument (Agilent Technologies) within 5 minutes after its preparation. Each library size was recorded in a Microsoft Excel sheet and the average library size measured was determined. The molarity of the library was calculated using the following formula [41]:

Molarity (nM) = [(ng/μL) × 10^6^]/[660 (g/mol) × average library size (bp)]

##### Dilution of the Library to the Starting Concentration

Samples (about 5 μL) were pipetted into separate microcentrifuge tubes. EB-0.1% Tween 20 mix was pipetted into each sample tube, where the volume varies so the final molarity of each sample was 4 nM. Next, 5 µL of each library was pooled into a single microcentrifuge tube. The library pool was subjected to Qubit to confirm the 4 nM concentration.

##### Sequencing

The Illumina iSeq 100 is a benchtop high-throughput sequencer, measuring 1 ft^3^ in size. This sequencer is useful for sequencing small genome, transcriptome, long amplicon, and targeted resequencing applications, generating up to 1.2 Gb data per run.

The cartridge was first thawed overnight in a water bath (20-25°C) in its foil bag. When the cartridge is ready, 4 nM library pool was diluted to 50 pM loading concentration using EB. About 10 nM of PhiX (control library) was diluted to 50 pM using EB. Then, 2 µL of PhiX (50 pM) was added to 98 µL library pool (50 pM), making the final pool volume as 100 µL.

The cover of the loading reservoir on the thawed cartridge was first teared using a pipette tip. Then, 20 µL final pooled library was pipetted into the bottom of the reservoir. Flow cell was inserted into the cartridge. Then, the cartridge-flow cell assembly was inserted into the iSeq 100 tray. Run was initiated using instrument control software. The total runtime was up to 17.5 hours.

#### Bioinformatics Analysis

The iSeq100 run monitoring was executed using BaseSpace Sequence Hub (Illumina) cloud-based software, where GRCh37 hg19 human reference assembly [42] was used to map genomic sequences. Variant calls and differential expression results for amplicon panels were produced using BaseSpace Sequence Hub and Local Run Manager (on-board).

The variant call format (VCF) files were extracted and downloaded from BaseSpace Sequence Hub. Variants with minor allele frequency of 0.05 or less, based on either the gnomAD or 1000 Genome database, were considered as rare gene mutations.

#### Variant Call Format Analysis

The VCF files were outsourced (BioEasy Sdn Bhd) for data cleaning and bioinformatics interpretation [43]. Gene filtering for *LDLR, APOB-100, PCSK9, LDLRAP1, ABCG5,* and *ABCG8* was conducted by coordination using the BED format file downloaded from the UCSC Genome Browser, and each VCF format file was subjected to gene filtering using BCFtools. The overlapping list of variants was merged into 1 single list of variants consisting of 1 sample per column using BCFtools. Consistent gene annotation was performed using the SnpEff and Genome Analysis Toolkit/GATK. The cumulative allele frequency and the global minor allele frequency (GMAF) values were annotated using BCFtools. REVEL scores for hg19 were prepared for annotation using BCFtools. The SIFT annotation was performed using the SIFT4G annotator program, while PolyPhen-2 annotation was prepared using BCFtools. Variants with no GMAF values and GMAF ≤5% were separated into different files.

#### Pathogenicity Interpretations

The cleaned and combined VCF files were further analyzed for variant pathogenicity using modified American College of Medical Genetics and Genomics guidelines [44,45]. The American College of Medical Genetics and Genomics guidelines classify variant pathogenicity based on the type of mutation and other clinical evidence such as medical history, population frequency, in silico prediction, functional study, and segregation data. Additional reliable sources of references were identified using ClinVar [46] and Leiden Open-source Variation Database [47] websites. The variants were ultimately classified into “pathogenic” (PV), “likely pathogenic,” “benign variant,” or “variant of unknown significance.” The RA, Genetic Laboratory staff, and the lipid specialists who were involved in analyzing and interpreting the genetic results were blinded to the index tests results (FAMCAT, SB, and DLCC) and the relevant variables collected in Work stream 1.

#### Delivery of the Genetic Results

The genetic analysis results for individual patients will be delivered by the clinician investigators. Patients who are genetically confirmed to have FH will be counseled regarding the nature of the genetic mutations, mode of inheritance, the need for pharmacological management, and cascade screening of first- and second-degree relatives. The importance of adherence to lifestyle modification and pharmacotherapy will also be emphasized. Lipid-lowering treatment for each patient will be reviewed and uptitrated to achieve an LDL-c target of <1.8 mmol/L [48,49]. Patients will also be supported psychosocially on how to adapt with the condition. They will be referred to the UiTM lipid specialists for further management and cascade screening of family members.

### Work Stream 3a: Exploring the Experience, Concern, and Expectation of Individuals With Suspected FH

#### Study Design, Setting, Population, and Method

This is a nested qualitative study performed in MOH primary care clinics. The study population included patients with suspected FH who have undergone genetic testing. Semistructured interviews and thematic analysis of data are performed.

#### Sampling Method

Patients with suspected FH who have received their genetic analysis results are purposefully sampled to reflect their social, ethnic, and educational diversity, and range of results including those who are genetically confirmed to have FH and those with negative results. Written informed consent is obtained from the participants.

#### Data Generation

Semistructured in-depth interviews are conducted face-to-face or virtually using the online platform Google Meet (Google LLC/Alphabet Inc.) by an RA, supervised by a Malaysian lead qualitative co-investigator (SAR) who is an expert in qualitative study methods. A topic guide developed for the interview (Multimedia Appendix 1) is used to help explore patients’ experience and concern of being identified with suspected FH, of undergoing FH genetic testing, and understand their experience of receiving the results and undergoing follow-up assessment and referral processes [50]. The interviews are conducted for up to 45 minutes, and they are audio- and video-recorded. Recruitment and data generation will continue until saturation of themes. This is expected to be achieved by interviewing approximately 10-15 patients [51,52].

#### Data Analysis

Data from the interviews will be transcribed verbatim and translated into English. Data will be organized using NVivo qualitative software (QSR International) [53], and analyzed thematically by JK, SAR, and LC. Coding and development of analysis will involve a minimum of 2 qualitative researchers (SAR and LC).

### Work Stream 3b: Evaluating Clinical Utility of the Web-Based FH Identification Tool in Malaysian Primary Care

#### Study Design, Setting, Population, and Method

This is a nested qualitative study conducted in MOH primary care clinics. The study population included PCPs working at the MOH Primary Care Clinics. The “think-aloud” interview method [54] is applied.

#### Sampling Method

PCPs are purposefully sampled to reflect their varied lengths of clinical experience, and range of use of the FAMCAT web-based FH Identification Tool in practice. Written informed consent is obtained from the participants.

#### Data Generation

Interviews are conducted using the “think-aloud” method [54] in which participants are required to talk aloud while using the FAMCAT web-based FH Identification Tool. A user manual and task scenarios (Multimedia Appendix 2; also see [26,55,56]) have been created to assist clinicians in performing clinical tasks simulation using the web-based tool. The clinical scenarios are standardized for all participants, regardless of their level of clinical experience. The task scenarios are reviewed by the research team members, including experienced and practicing PCPs, to ensure that the content, format, and presentation are representative of real clinical use and addressed the major functional components of the application.

The “think-aloud” interview method generates direct data on participants’ ongoing thought processes during task performance. The underlying assumption of “think aloud” is based on human cognition where it is postulated that only information capable of being verbalized in situ is being actively processed in the working memory and so is taken as an active representation of task performance [54]. The output of this process is called a verbal protocol, which is then transcribed and systematically analyzed to develop a model of the persons’ task behavior.

Video-based observational data are collected to record the participants’ interaction with the interface and functionality of the FAMCAT web-based FH Identification Tool. These data are time-locked for analysis to the verbal protocols. Following the end of the task simulations, participants complete a short questionnaire to capture a subjective measure of their user satisfaction with the interface and functionality of the FAMCAT web-based FH Identification Tool. Recruitment and data generation will continue until saturation of themes and this is expected to be achieved by interviewing approximately 10-15 participants [51,52].

### Data Analysis

Each verbal protocol will be transcribed and coded using a predefined coding scheme following the categories of interest. Similarly, the audio-video–recorded simulation will provide a range of images on all of the tasks including time to completion, types of clinical knowledge, resources accessed, participants’ statements, and the tasks associated therewith. Two qualitative researchers will independently map the statements made by participants and the video observations, identifying both “positive” and “negative” mental heuristics; the former demonstrating potential features that are acceptable to the needs of participants, and the latter to determine whether there are any possible refinements that might be made to the FH Identification Tool.

### Ethics Approval

This study protocol was approved by the respective research ethics committees in Malaysia, namely, the UiTM Research Ethics Committee [(REC/03/2020) (FB/48)] and the Medical Research Ethics Committee of the Ministry of Health Malaysia [NMRR-20-272-52797 (IIR)].

## Results

The recruitment for Work stream 1 and blood sampling and genetic analysis for Work stream 2 were completed in February 2023. The data collection for Work stream 3a and 3b was completed in March 2023. Data analysis for Work streams 1, 2, 3a, and 3b is projected to be completed by June 2023, with the results of this study anticipated to be published by December 2023.

## Discussion

### Expected Findings

To the best of our knowledge, this is the first study to assess the diagnostic accuracy of different clinical diagnostic criteria (SB, DLCC, and FAMCAT) in identifying FH in the Malaysian primary care setting, with molecular diagnosis targeting the 4 FHCGs (*LDLR, APOB-100, PCSK9,* and *LDLRAP1*) as the gold standard. The findings will also include molecular analysis of the 2 HCAGs (*ABCG5* and *ABCG8*). The full spectrum of genetic mutations in these 6 genes including novel PVs will be identified. This study also included nested qualitative research exploring the experience, concern, and expectation of patients with suspected FH who have undergone genetic testing and also real-time observation of PCPs using the “think-aloud” method to evaluate the clinical utility of the FAMCAT web-based FH Identification Tool in the Malaysian primary care setting. In a nutshell, it is a comprehensive mixed methods study that uses both quantitative and qualitative research methods to answer complex research questions, understand the process, and strengthen the impact evaluation [31].

A previous diagnostic accuracy study that included 755 individuals with an LDL-c level of 4.0 mmol/L or more from secondary care clinics and community health screenings in Malaysia showed that the SB criteria appeared to be the most useful in identifying FH compared with the Japanese FH Management Criteria and the US Make Early Diagnosis to Prevent Early Deaths criteria, when assessed against the DLCC as the gold standard [57]. However, unlike this study, genetic testing was not performed in the previous study.

Recent studies have shown that NGS is the method of choice [58,59] and is cost-effective in the genetic diagnosis of FH in primary care [60-62]. The targeted NGS method [37] that is being used in this study is robust and efficient in terms of speed, read length, and throughput, designed to identify the 4 FHCGs and the 2 HCAGs, giving an overall gene coverage of 98.61% [38-40].

The nested qualitative research exploring the patients’ perspectives while undergoing genetic testing and the PCPs experience in utilizing the web-based tool will add tremendous value in developing a clinical care pathway that is patient-centered [63]. A recent qualitative study [64] showed that the main benefit of genetic diagnosis from the patients’ perspective lies in its ability to provide accurate information to their younger family members to undergo screening, and subsequently receive timely management to reduce their risk of having premature CAD. In the United Kingdom, patients and practitioners reported a positive experience when genetic testing was introduced with electronic case-finding for FH in primary care [63].

### Implications on Clinical Practice

The findings from this study will be shared through scientific publications, conference presentations, and through a resource impact report with the key stakeholders. The resource impact report will include a patient-centered clinical care pathway that will be implemented by the stakeholders in primary care. FH management training modules for primary care providers will also be developed and delivered through online webinars or face-to-face workshops, to enhance their knowledge and skills in managing FH. Dissemination of knowledge regarding FH and its genetic causes will also be made available to the general public through public health campaigns using various mass media channels and social media platforms. All of these imperative measures will accelerate early FH identification in the Malaysian primary care setting and subsequently reduce the risk of premature CAD.

### Limitations

This study has several limitations. First, the selection of sites was limited to the clinics located in the urban and suburban conurbations that can access genetic testing facility at the I-PPerForM Genetic Laboratory. Future research should include a wider selection of clinics including those in the rural parts of Malaysia. Second, only those clinics with EMR were invited to participate as the screening for patients with elevated LDL-c levels was conducted through the EMR. This could potentially cause selection and recruitment bias. To minimize this bias, purposive sampling was conducted by targeting those with the highest LDL-c levels, with study invitations sent to all patients in the highest LDL-c groups of 7.0 mmol/L or more and 6.0-6.9 mmol/L, while an equal number of patients were invited from the lower LDL-c groups of 5.0-5.9 mmol/L and 4.0-4.9 mmol/L.

Third, this study will provide insights into the nature of mutations in the 6 targeted genes, but it does not include functional studies to validate the pathogenicity of the potential PV, which is warranted in future studies. The polygenic hypercholesterolemia genes were also not included as they are not within the scope of this study. Therefore, future studies are warranted to evaluate the impact of polygenic hypercholesterolemia genes on patients with clinically suspected FH without any PV. Additionally, the targeted NGS method used in this study was not able to detect large gene rearrangements, which may account for a small proportion of PV in patients with FH [65,66].

Fourth, the molecular analysis was performed only in index cases. Cascade screening of family members will be conducted and they will be clinically diagnosed with SB or DLCC. Unfortunately, we are not able to offer genetic testing to the family members due to financial constraints and this is also beyond the scope of this study.

### Conclusions

This comprehensive mixed methods study will provide objective evidence on which clinical diagnostic criterion is the best to detect FH in the Malaysian primary care setting against molecular diagnosis as the gold standard. The full spectrum of genetic mutations in the 6 targeted genes including novel PVs will be identified. Patients’ perspectives while undergoing genetic testing and the PCPs experience in utilizing the web-based tool will be established in this study. These findings will have a tremendous impact in advancing the scientific knowledge and skills on the clinical and genetic diagnosis of patients with FH in primary care and subsequently reduce their risk of premature CAD.

## Appendix

Multimedia Appendix 1 Topic guide to explore the experience, concerns and expectations of individuals with suspected Familial Hypercholesterolaemia.

Multimedia Appendix 2 The FAMCAT Web-Based Familial Hypercholesterolaemia Identification Tool user manual and task scenarios for primary care physicians.

Multimedia Appendix 3 MRC UK peer-review reports and grant offer letter.

## Abbreviations

ABCG5: ATP-binding cassette subfamily G member 5
ABCG8: ATP-binding cassette subfamily G member 8
APOB-100: apolipoprotein B-100
APOE: apolipoprotein E
CAD: coronary artery disease
DLCC: Dutch Lipid Clinic Criteria
EB: elution buffer
EMR: electronic medical record
FAMCAT: Familial Hypercholesterolemia Case Ascertainment Tool
FH: familial hypercholesterolemia
FHCG: familial hypercholesterolemia candidate gene
FMS: family medicine specialist
GMAF: global minor allele frequency
HCAG: hypercholesterolemia-associated gene
HeFH: heterozygous familial hypercholesterolemia
HoFH: homozygous familial hypercholesterolemia
LDL-c: low-density lipoprotein cholesterol
LDLR: low-density lipoprotein receptor
LDLRAP1: low-density lipoprotein receptor adaptor protein 1
MOH: Ministry of Health
NGS: next-generation sequencing
PCP: primary care physician
PCSK9: proprotein convertase subtilisin/kexin type 9
PV: pathogenic variant
RA: research assistant
SB: Simon Broome
UiTM: Universiti Teknologi MARA
VCF: variant call format

## Data Availability

Raw data are kept at the Institute of Pathology, Laboratory and Forensic Medicine (I-PPerForM), Universiti Teknologi MARA, Selangor, Malaysia and encrypted data are kept at the University of Nottingham, United Kingdom in secured databases. Unidentified data will be shared by the corresponding authors upon request after all the results of this study have been published; and the data are subjected to the data protection regulations of Malaysia and the United Kingdom.
