# Supplementary material for: Reducing Premature Coronary Artery Disease in Malaysia by Early Identification of Familial Hypercholesterolemia Using the Familial Hypercholesterolemia Case Ascertainment Tool (FAMCAT): Protocol for a Mixed Methods Evaluation Study
Source: JMIR Res Protoc. 2023 Jun 2;12:e47911. doi: 10.2196/47911 (PMC10276320; doi:10.2196/47911)
Supplement: Multimedia Appendix 1 [file resprot_v12i1e47911_app1.pdf]

INTRODUCTION

Good morning Mr./Mrs./Ms.____________. My name is___________________________.

Thank you for taking the time to be interviewed today.

WARM-UP CHAT (UNSTRUCTURED):

As I have described in the text message or phone call, our research team is currently working to explore the experience, concern, and expectation of patients undergoing genetic testing for Familial Hypercholesterolaemia (FH). You are being invited to participate in this interview because you recently participated in a study titled "Reducing Premature Coronary Artery Disease by Early Identification of Familial Hypercholesterolaemia". You were identified to have a high risk of FH and your blood sample has been sent for genetic testing.

This interview will explore your experience, concern, and expectation when you were informed that you were identified to be at risk for FH and were undergoing the genetic testing. It will last approximately 45 minutes to 1 hour. This interview will be audio and video-recorded.

You have agreed to be interviewed and have signed the consent form to participate in this study. You have also agreed to be audio and video-recorded. Before we start, is there any question or further clarification that you would like me to address?

*Give time for patients to respond.

I shall now start the audio and video recording.

We shall start the interview. I am going to ask you several questions, and I appreciate if you could answer each question as honestly as possible.

ATTITUDE & BELIEF

1. What is your perception of genetic testing for FH? – Expectation
2. Can you tell me what is your overall opinion on genetic testing for FH? – Belief
3. Why do you think it was necessary/not necessary to do it? – Expectation
4. Do you think that genetic testing for FH is important? If yes, why. If no, why. – Belief
5. Do you think that genetic testing for FH should have been done sooner or later? – Expectation
6. Do you think having a genetic testing is against your religion or beliefs? Please explain. – Belief
7. Do you have any plan after you are informed of the genetic test results? – Expectation
8. In your opinion, how accessible is genetic testing for FH in this country? If yes, why. If no, why. – Belief
9. Do you think the test should be made widely available/accessible? If yes, why. If no, why. – Expectation
10. Would you have agreed to take this test if you have to pay for the genetic testing? – Expectation
11. Would you recommend genetic testing for FH to someone you know that may have the same personal or family history like you do? – Experience

INFORMATION AND SUPPORT

1. What sort of information was given to you during the pre-test counselling for genetic testing? – Experience
2. Do you think the information given are sufficient and helpful? If yes, why. If no, why. – Experience
3. Do you wish that the pre-test counselling was done differently? – Expectation
4. Was there any information that you wished was relayed better? – Expectation
5. What kind of information that you expect to have before considering to take up the genetic testing? – Expectation

6. How would you like to receive the information about the genetic testing? – Expectation

7. Since FH is new to you, how would you like to receive information about the condition? – Expectation

8. Do you think that the results of genetic testing for FH is important for you to make healthcare decision? Please give your opinion on this - Experience

RETURN OF RESULTS

1. How do you feel when you were waiting to receive the genetic test results? – Experience, concern

2. What do you anticipate from your results? – Expectation, concern

3. How do you feel when you received your genetic test results? – Experience, concern

4. How would you prefer to have your results delivered? - Expectation
   (Prompts: by a geneticist, by a specialist, by mail etc.)

5. Would you let your family members know of your genetic test results? – Experience, concern

6. Is there any shortcoming that you would like to address to improve the 'return of your genetic test results'? – Expectation, concern

ETHICAL & PRIVACY CONCERNS

1. Do you have any ethical and privacy concerns related to the genetic testing for FH? If yes, please elaborate. – Expectation, concern

2. How do you feel about having the medical profession know of your condition? – Expectation, concern

3. Were you given a chance to give an informed consent prior to agreeing to undergo the genetic testing for FH? – Experience

FAMILY COMMUNICATION

1. How do you feel about having to tell your genetic test results to your family members? – Experience

2. Since your results is positive for FH, how do you feel about inviting your family members to be screened for FH? – Experience

(This question is only for those with positive results)

3. Do you need assistance from health professionals to break the news to your family members? – Expectation

4. Would you recommend genetic testing for FH to your family members (offspring, siblings, close relatives)? – Experience, Expectation

---

This interview has come to the end. Thank you for taking the time to respond to all of the questions.
