# Supplementary material for: Reducing Premature Coronary Artery Disease in Malaysia by Early Identification of Familial Hypercholesterolemia Using the Familial Hypercholesterolemia Case Ascertainment Tool (FAMCAT): Protocol for a Mixed Methods Evaluation Study
Source: JMIR Res Protoc. 2023 Jun 2;12:e47911. doi: 10.2196/47911 (PMC10276320; doi:10.2196/47911)
Supplement: Multimedia Appendix 2 [file resprot_v12i1e47911_app2.pdf]

INTRODUCTION

The FAMCAT Web-Based Familial Hypercholesterolaemia Identification Tool was developed to assist primary care physicians in identifying individuals who may have Familial Hypercholesterolaemia (FH). The development of this web-based tool was funded by the Newton-Ungku Omar Fund (NUOF): The UK-Malaysia Joint-Partnership Call on Non-Communicable Diseases - Reducing Premature Coronary Artery Disease by Early Identification of Familial Hypercholesterolaemia, Grant reference: 100-TNCPI/GOV 16/6/2 (002/2020)-02 and MR/T 017384/1.

This web-based tool contains the newly developed Familial Hypercholesterolaemia Case Ascertainment Tool (FAMCAT) [1] and two established FH clinical diagnostic criteria i.e. Simon Broome (SB) [2] criteria and Dutch Lipid Clinic Criteria (DLCC) [3]. Using this tool, individuals who may have FH can be clinically diagnosed and offered genetic testing if resources are available.

INSTRUCTIONS

Please navigate through this link to access the FAMCAT Web-Based Familial Hypercholesterolaemia Identification Tool: https://ilixa.com/famcat/

This link will take you to the front page of the web-based tool:

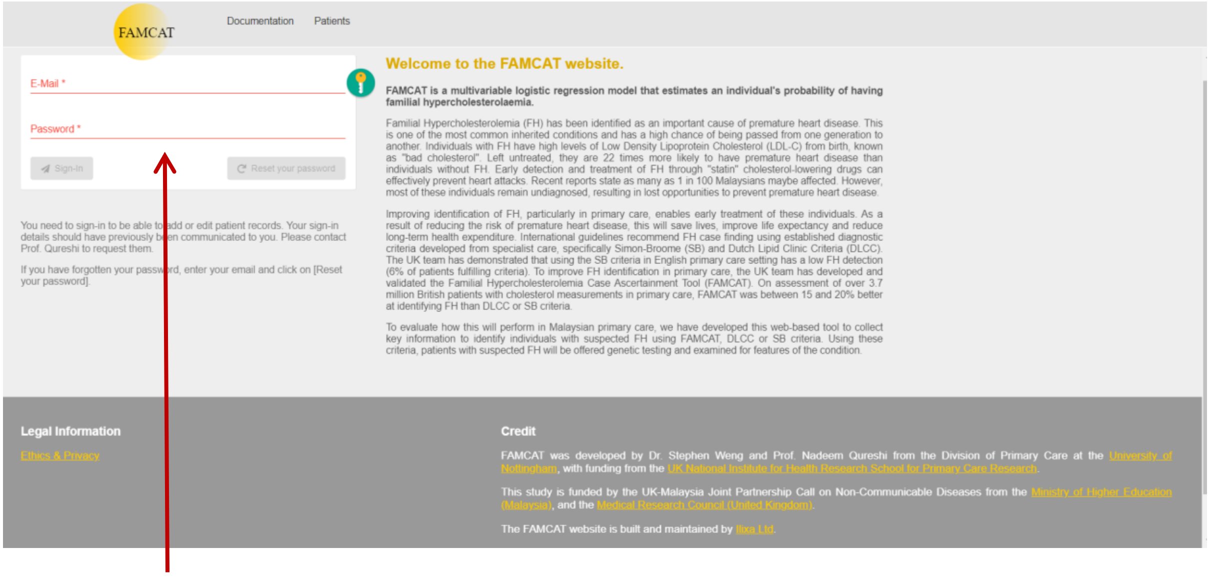

Please log in using the email address and password provided.

Click sign in. The same page will appear as below:

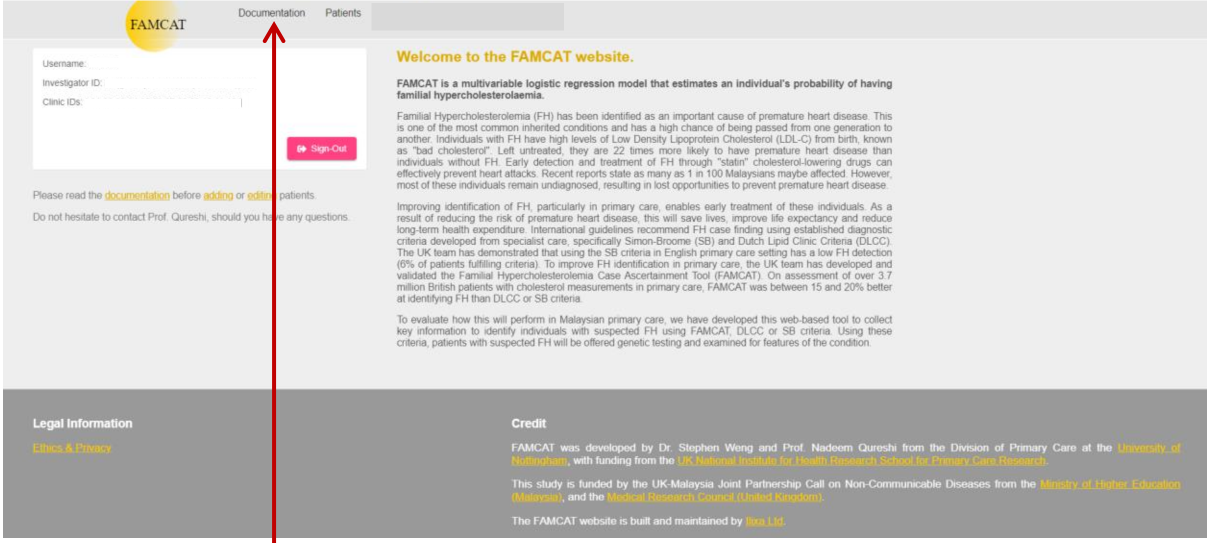

Please click the ‘**Documentation**’ tab to get more information about this project.

This will take you to the ‘**Documentation**’ page of the web-based tool:

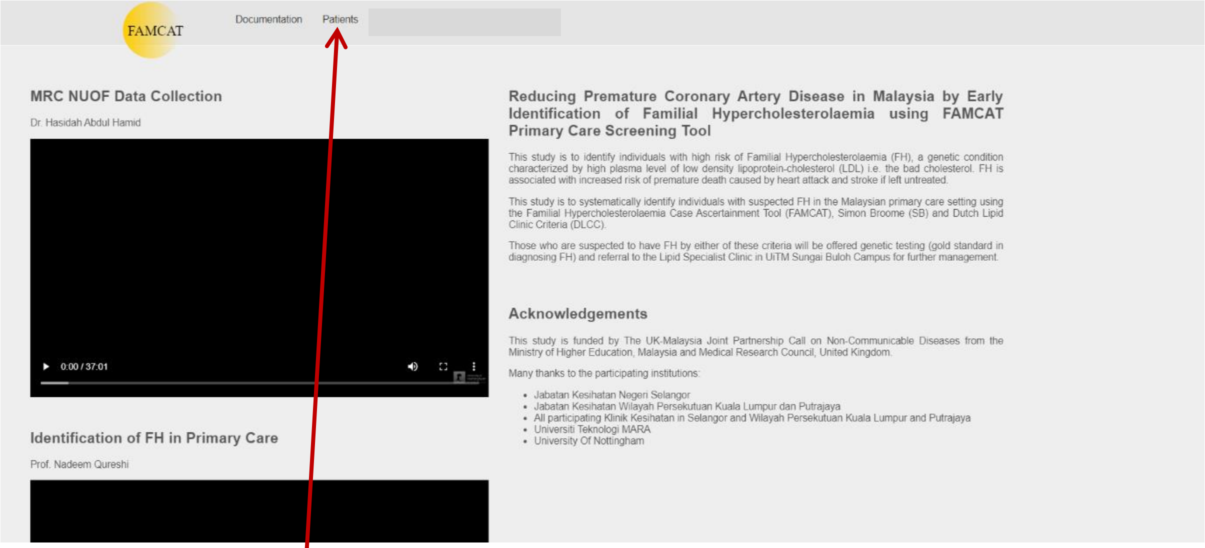

Please watch the two videos on this page for information regarding FH identification in primary care.

Please click the ‘**Patients**’ tab to start using the FAMCAT Web-Based Familial Hypercholesterolaemia Identification Tool. This will take you to the following page:

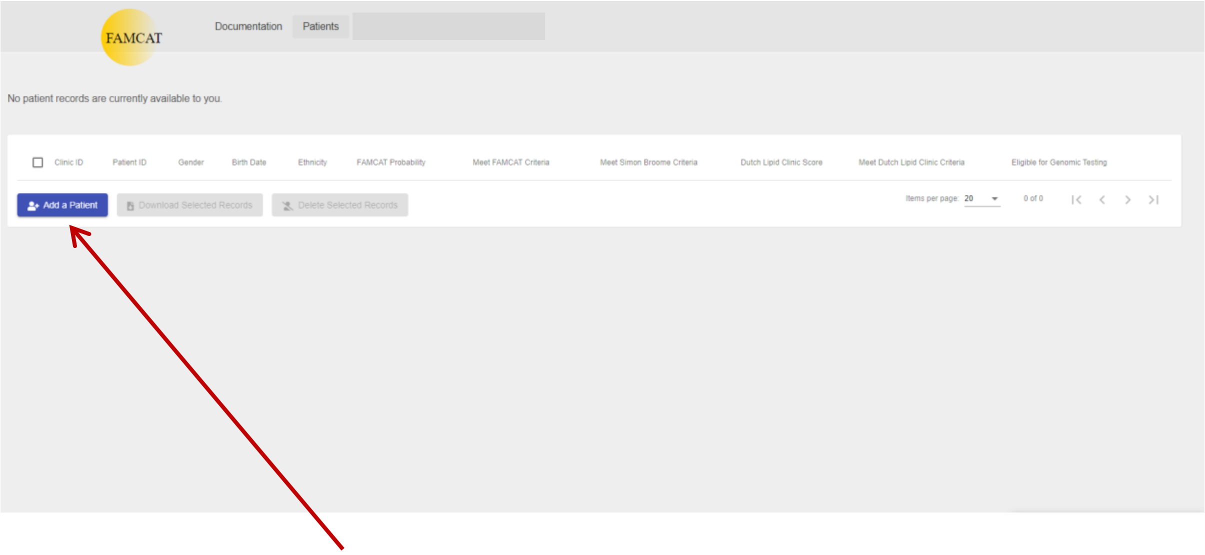

Please click on the ‘**Add a Patient’** tab.

This will take you to the following page:

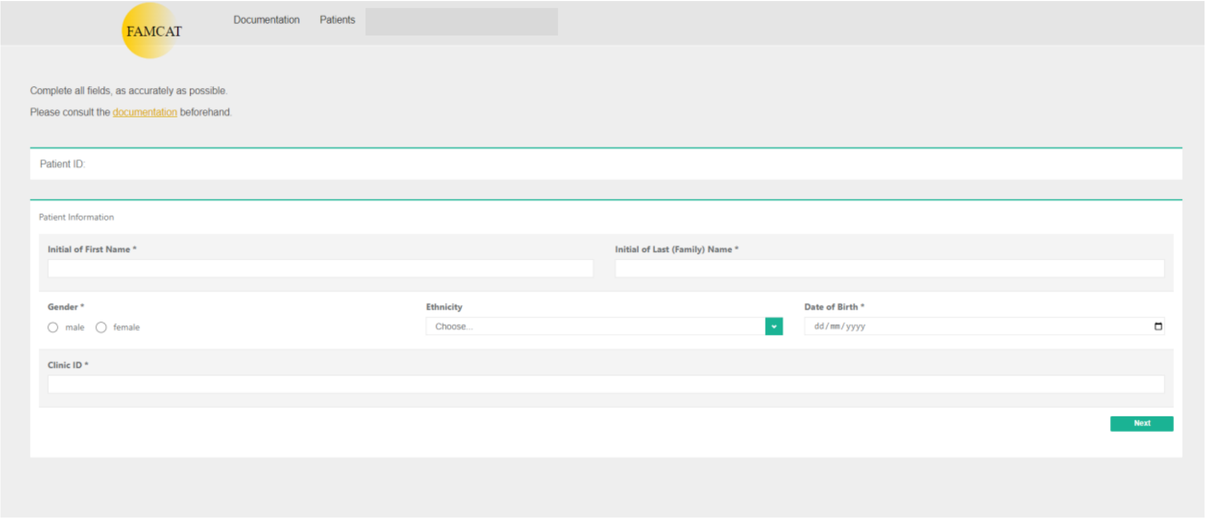

You are given three clinical scenarios which you may use to practice entering clinical data into the web-based tool.

Please read the following scenario:

TASK SCENARIO 1

CASE 1

Clinic ID	○ **09**
Name	○ **Dariah Binti Mansor**
Ethnicity	○ **Malay**
Gender	○ **Female**
D.O.B	○ **27.02.1967**
Age	○ **56 Years old**
The highest Fasting Lipid Profile: 14.10.2017	○ **Total cholesterol: 11.9 mmol/L**<br>○ **LDL-c: 9.39 mmol/L**<br>○ **Triglyceride: 2.3 mmol/L**<br>○ **HDL-c: 1.45 mmol/L**
Lipid Lowering Medication	○ **No medication**
Personal Medical History	○ **Hypertension, Heart Failure, Hypercholesterolaemia**
Family Medical History	○ **Father had myocardial infarction (MI) at the age of 45 years old.**<br>○ **Uncle had MI in his late 40s**<br>○ **No known history of very high cholesterol level among family members**<br>○ **No family member has been genetically diagnosed with Familial Hypercholesterolaemia**
Physical Examination	○ **Corneal arcus in both eyes since the age of 40 years old**<br>○ **Tendon xanthomata found on both hands, knees and ankles**
Additional Family History	○ **Mother had tendon xanthomata and corneal arcus at a young age**

Physical Examination

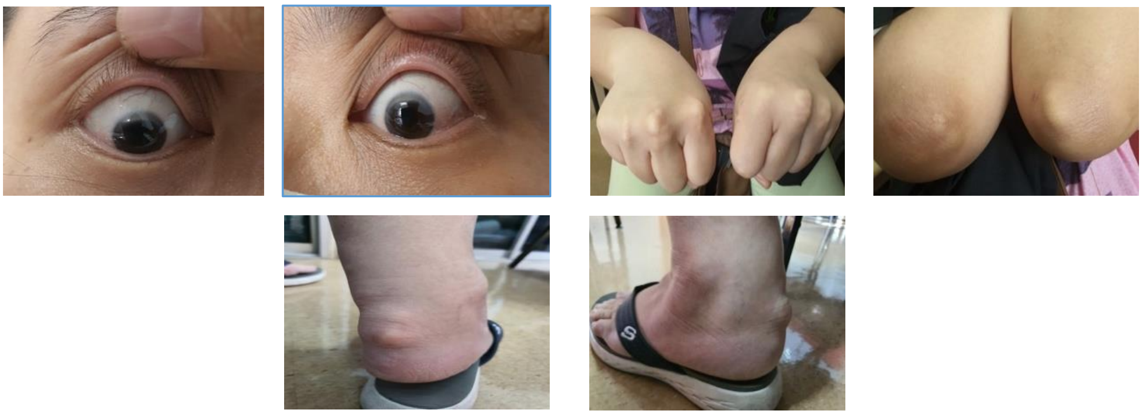

Task 1: Using Task Scenario 1, please enter patient's personal details on this '**Patient Information**' page:

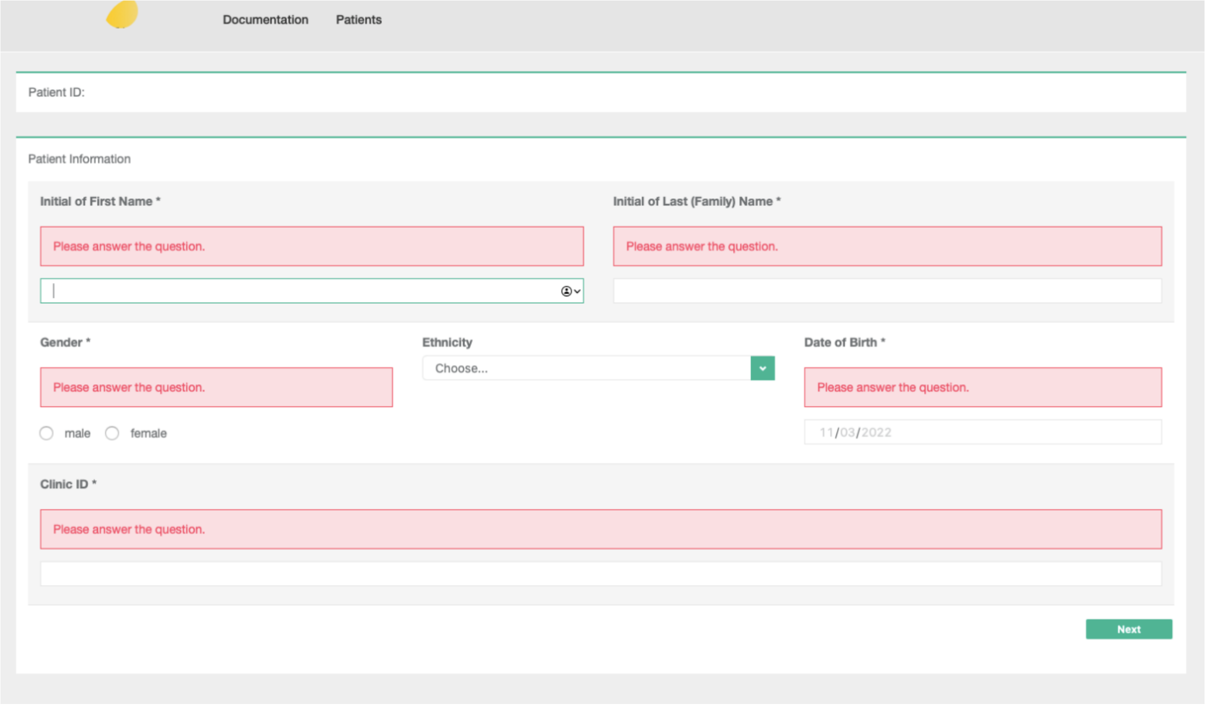

Press the '**Next**' tab once you have finished entering the information.

Task 2: Using the same scenario, please enter patient's cholesterol readings into this '**Cholesterol**' page:

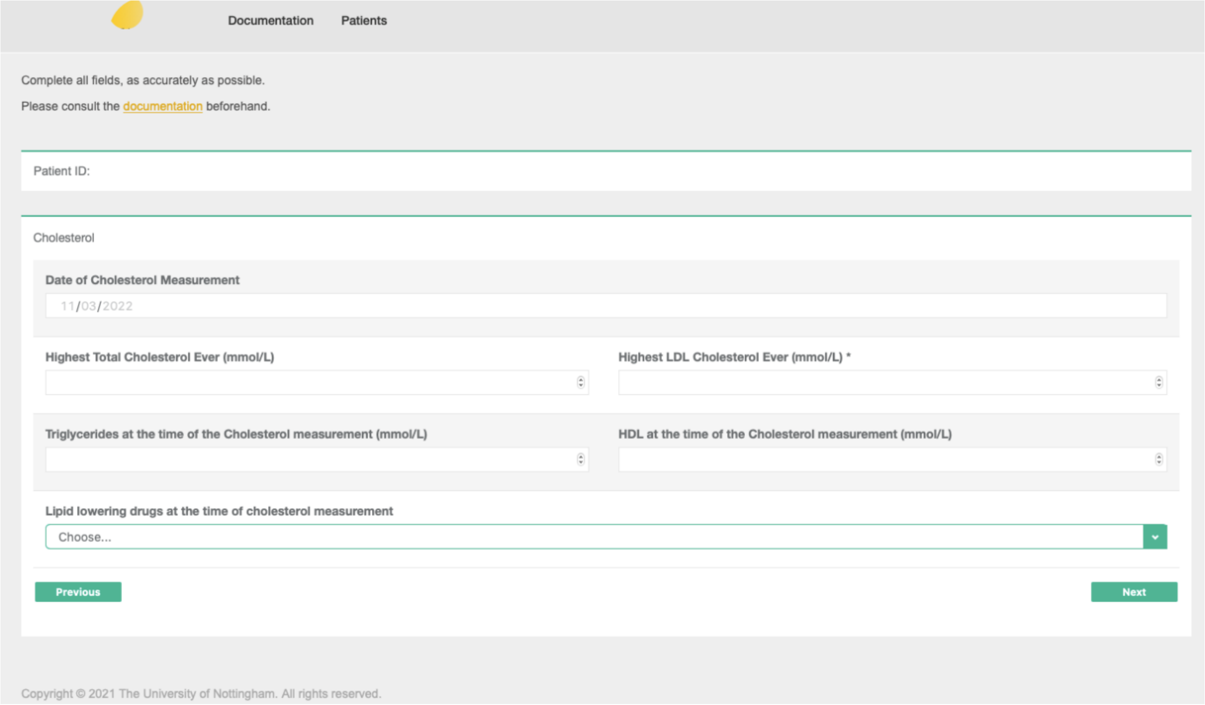

Press the ‘**Next**’ tab once you have finished entering the information.

Task 3: Using the same scenario, please enter patient’s personal medical history and family history into the ‘**History**’ page:

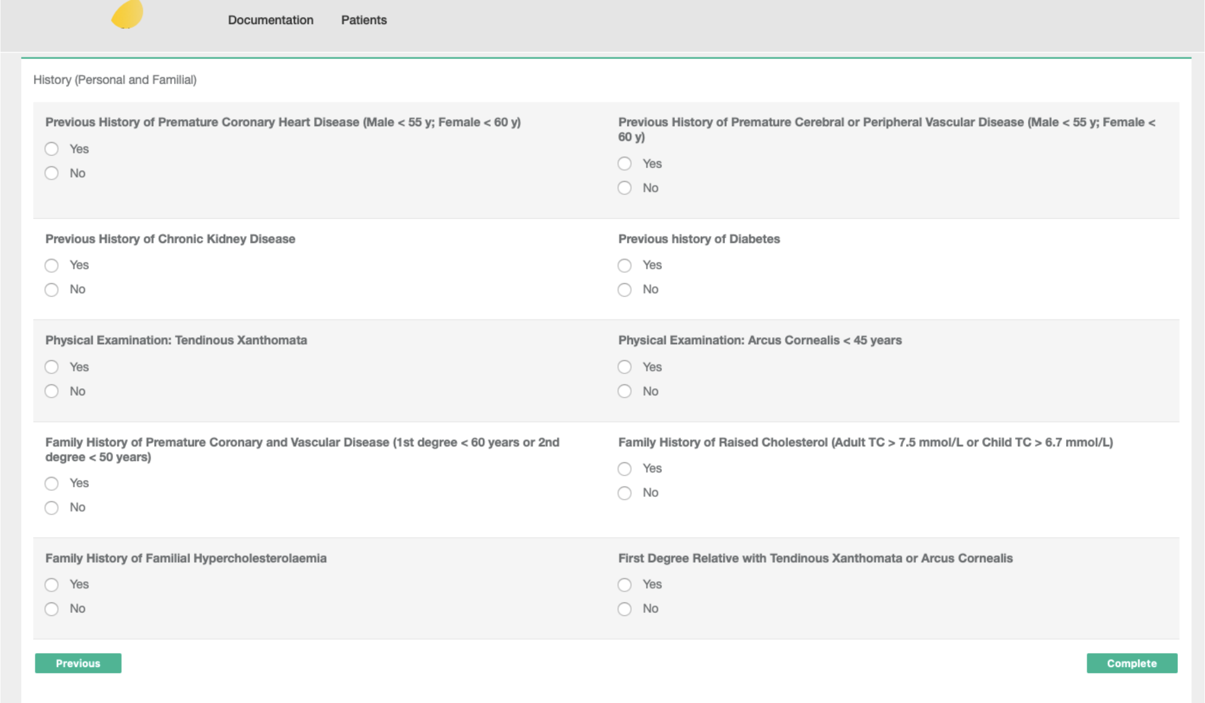

Press the ‘**Complete**’ tab once you have finished entering the information.

The following message will appear:

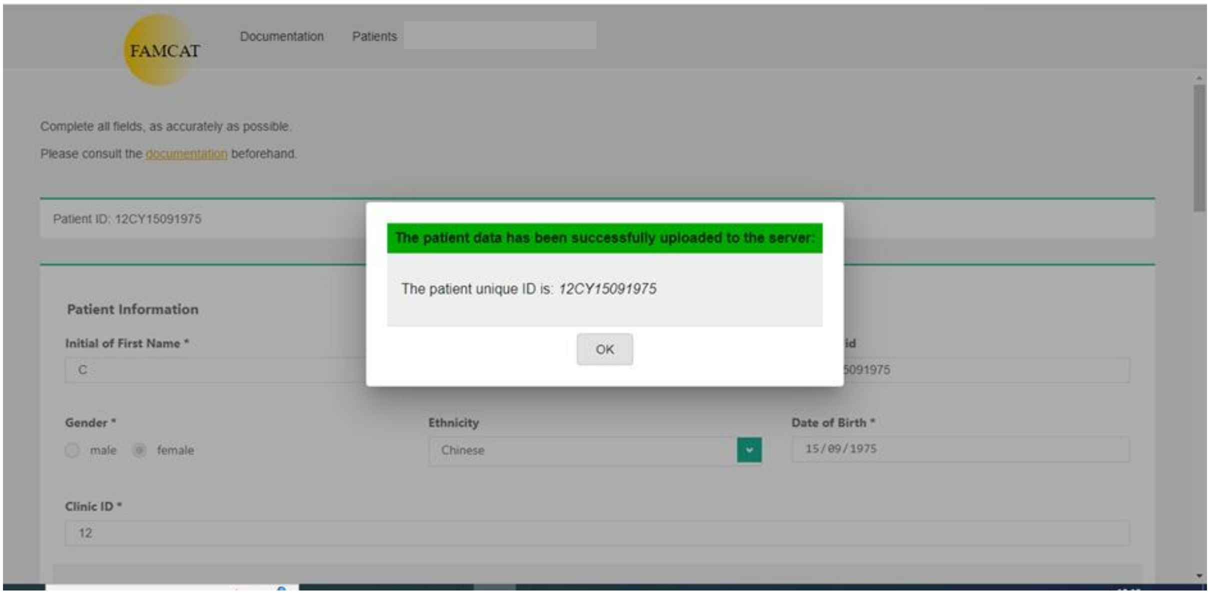

Press the ‘**OK**’ tab, and the patient’s summary page will appear as shown in the next page.

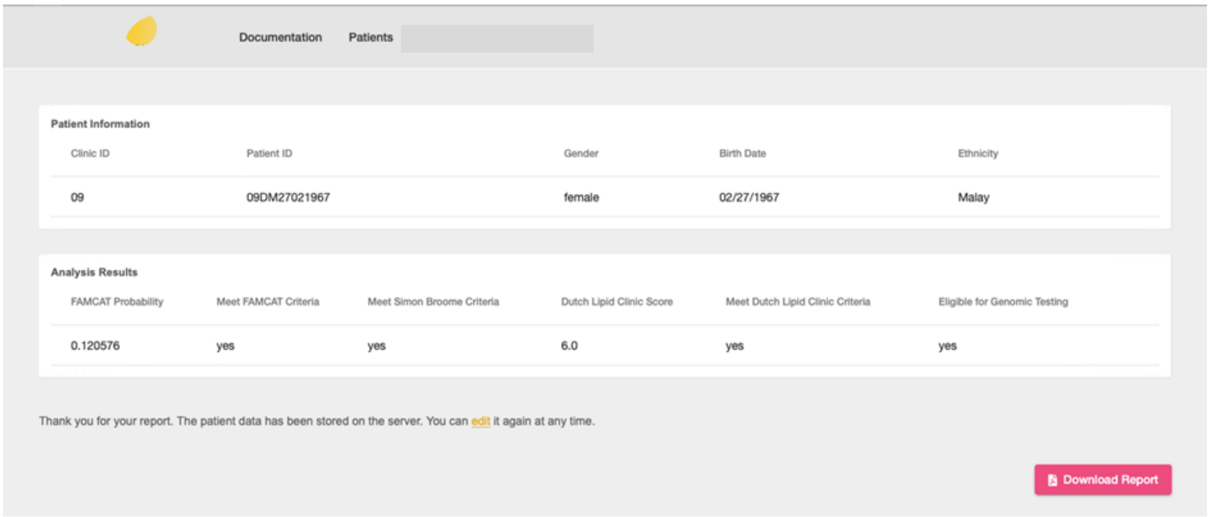

On this summary page, you can see your patient’s brief details, which diagnostic criteria that your patient fulfilled and whether this patient is eligible to be offered genetic testing.

Please click the ‘**Download Report**’ tab to produce a PDF summary of the patient that you have entered.

Please repeat the same procedure for patients in the Task Scenarios 2 and 3.

TASK SCENARIO 2

CASE 2

Clinic ID	○ **10**
Name	○ **Muhammad Firdaus Bin Othman**
Ethnicity	○ **Malay**
Gender	○ **Male**
D.O.B	○ **26.08.1985**
Age	○ **38 years old**
The highest Fasting Lipid Profile: 14.10.2017	○ **Total cholesterol: 7.3 mmol/L**<br>○ **LDL-c: 5.1 mmol/L**<br>○ **Triglyceride: 1.6 mmol/L**<br>○ **HDL-c: 1.5 mmol/L**
Lipid Lowering Medication	○ **No medication**
Personal Medical History	○ **Gastritis**
Family Medical History	○ **Father had myocardial infarction (MI) at the age of 45 years old**<br>○ **Paternal grandfather had MI and died at the age of 59 years old**<br>○ **No known history of very high cholesterol level among family members**<br>○ **No family member has been genetically diagnosed with Familial Hypercholesterolaemia**
Physical Examination	○ **Corneal arcus in both eyes since the age of 36 years old**<br>○ **There is no tendon xanthoma**

Physical Examination

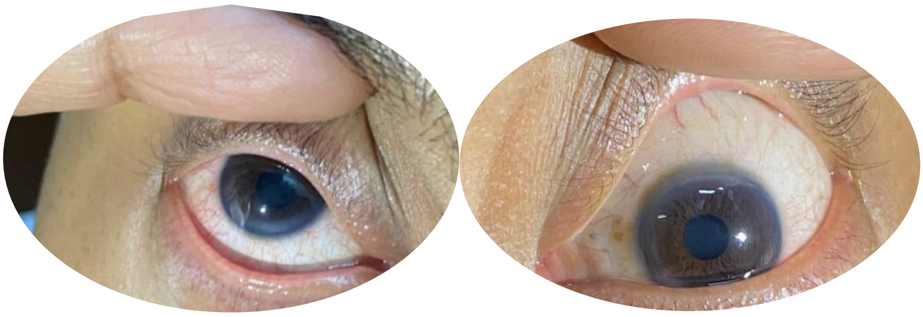

TASK SCENARIO 3

CASE 3

Clinic ID	○ **11**
Name	○ **Munianmal A/P Superaniam**
Ethnicity	○ **Indian**
Gender	○ **Female**
D.O.B	○ **19.05.1971**
Age	○ **52 Years old**
The highest Fasting Lipid Profile: 14.10.2017	○ **Total cholesterol: 7.3 mmol/L**<br>○ **LDL-c: 5.1 mmol/L**<br>○ **Triglyceride: 1.6 mmol/L**<br>○ **HDL-c: 1.5 mmol/L.**
Lipid Lowering Medication	○ **Simvastatin 40mg**
Personal Medical History	○ **Asthma**
Family Medical History	○ **Father had myocardial infarction (MI) at the age of 45 years old**<br>○ **Known history of very high cholesterol level among family members**<br>○ **No family member has been genetically diagnosed with Familial Hypercholesterolaemia**
Physical Examination	○ **Corneal arcus in both eyes since the age of 50 years old**<br>○ **There is no tendon xanthoma**

Physical Examination

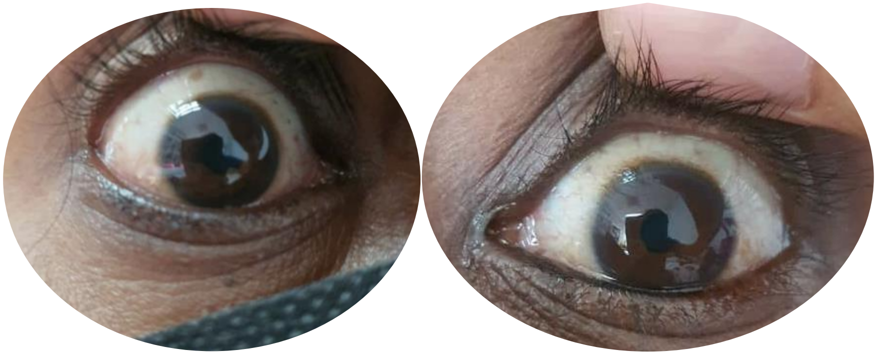

REFERENCES

1. Weng SF, Kai J, Neil HA, Humphries SE, Qureshi N. Improving identification of familial hypercholesterolaemia in primary care: Derivation and validation of the familial hypercholesterolaemia case ascertainment tool (FAMCAT). Atherosclerosis. 2015;2:336-43.
2. Mortality in treated heterozygous familial hypercholesterolaemia: implications for clinical management [Scientific Steering Committee on behalf of the Simon Broome Register Group]. Atherosclerosis 1999; 142:105-12.
3. Watts GF, Sullivan DR, Poplawski N, et al. Familial hypercholesterolaemia: a model of care for Australasia. Atheroscler Suppl 2011; 12:221-63.
