# Supplementary material for: Reducing Premature Coronary Artery Disease in Malaysia by Early Identification of Familial Hypercholesterolemia Using the Familial Hypercholesterolemia Case Ascertainment Tool (FAMCAT): Protocol for a Mixed Methods Evaluation Study
Source: JMIR Res Protoc. 2023 Jun 2;12:e47911. doi: 10.2196/47911 (PMC10276320; doi:10.2196/47911)
Supplement: Multimedia Appendix 3 [file resprot_v12i1e47911_app3.pdf]

Medical Research Council
2nd Floor David Phillips Building, Polaris House, North Star Avenue, Swindon,
United Kingdom SN2 1ET
Telephone +44 (0) 1793 416200
Web http://www.mrc.ac.uk/

COMPLIANCE WITH THE DATA PROTECTION ACT 1998

In accordance with the Data Protection Act 1998, the personal data provided on this form will be processed by MRC, and may be held on computerised database and/or manual files. Further details may be found in the **guidance notes**

Research Grant Peer Review

MRC Reference: MR/T017384/1

Document Status: With Council

UK-Malaysia Health Research Partnership

2019

Applicant Details

Applicant	Professor Nadeem Qureshi	Organisation	University of Nottingham

Title of Research Project

Reducing Premature Coronary Artery Disease in Malaysia by early identification of Familial Hypercholesterolaemia

Review Information

Response Due Date	09/08/2019	Reviewer Reference:	079046588

Research Quality

Research Quality: Please comment on the importance and competitiveness of the proposed research, including:

(1) strength of medical or scientific case (2) level of innovation, and whether this is likely to lead to significant new understanding (3) management strategy proposed, including equitable access to any shared resources (4) feasibility of experimental plans, statistics, methodology and design, including provision of sample size calculations, strategies to avoid bias, and preliminary data where appropriate (5) how well risks have been identified, and will be mitigated.

1. Strength of medical or scientific case: FH is prevalent in Malaysia and an important cause of premature CAD. There is need for greater awareness, detection and control of FH in that country. The proposed collaboration between UK and Malay investigators will help to reduce the burden of FH and premature CAD.

(2) level of innovation, and whether this is likely to lead to significant new understanding. Innovative aspects include a comparison of various criteria for FH in the Malay population, new knowledge about the genetic etiology of FH in Malays, creation of a web tool for FH case identification in the Malay primary care setting and its acceptability by care providers, as well as the patient experiences in detection and genetic testing for FH.

(3) Management strategy proposed is well thought out with equitable access to shared resources.

(4) The experimental plans, statistics, methodology and design are feasible and well crafted. The considerable prior experience of PI in setting up similar studies in UK and as part of NICE show in the design of the proposal. The sample size calculations, strategies to avoid bias, and preliminary data are satisfactory.

(5) Potential hurdles and plans to address these have been outlined.

Some limitations include:
1) The familial sharing of FH diagnosis and genetic testing results should have been dealt with in greater detail.
2) Similarly the plan for variant annotation is skeletal. More detail should have been presented. It is unclear where the variant frequencies will be obtained from and whether that source is relevant to Malay population.
3) Since the source of cases is the EMR, the investigators should consider clinical decision support integration into the EMR to help primary care providers manage patient at point of care.
4) For case identification, there should a step where those with secondary causes of elevated cholesterol should be excluded

Research Environment and People

Please comment on the suitability of the investigator group and the environment where the proposed research will take place, including (1) track record(s) of the individuals in their field(s) and whether they are best-placed to deliver the proposed research (2) level of commitment of host research organisation to supporting the proposed research (3) whether appropriate facilities will be available to the researchers

The investigative group is strong. The PI Dr. Nadeem Qureshi has a track record of research in FH. The investigators in Malaysia also have experience in chronic disease prevention and FH. Together the group is well positioned to accomplish the proposed aims. The host organizations are supportive of the research proposal as evidenced by the letters enclosed. Appropriate facilities are available to the researchers.

Impact

Please comment on the potential economic and societal impact of the proposed research, including (1) identification of realistic potential improvements to human or population health (2) contribution to relieving disease/disability burden and/or improving quality of life (3) identification of potential impacts of research and plans to deliver these (in the Pathways to Impact statement)

The proposal has the potential to improve population health in Malaysia by early detection and treatment of FH thereby reducing the burden of FH in the community. By creating tools to increase awareness, detection, treatment and control of FH in primary care in Malaysia, the project can have significant impact, given the high prevalence of FH in that country

Ethics

Please comment on any ethical and/or research governance issues, including (1) whether proposed research is ethically acceptable (2) any ethical issues that need separate consideration (3) appropriateness of ethical review and research governance arrangements (4) any potential adverse consequences for humans, animals or the environment and whether these risks have been addressed satisfactorily in the proposal

There are no ethical concerns regarding the project and the ethical review and research governance arrangements are acceptable. Any potential adverse consequences for humans have been addressed. However greater emphasis should be placed on the means for cascade testing including the ethical aspects of contacting family members of FH patients.

Data Management Plan

Please assess whether the data management plan indicates whether the applicants have (or are likely to have) a sound plan for managing the research data funded through the award, taking into account (1) the types, scale and complexity of data being (or to be) managed; (2) the likely long-term value for further research including by sharing data; and (3) the anticipated information security and ethics requirements.

The plan of managing research data is sound and appropriate human subjects protections are in place. Several types of datasets will be generated by the study and these will be a useful resource for the current and future investigators generating long term values.

Resources Requested

Please comment on (1) whether funds requested are essential and justified by the importance and scientific potential of the research (2) investigator time and proposed involvement related to management of the research (3) whether the proposal demonstrates value for money in terms of the resources requested (4) whether any animal use is fully justified in terms of need, species, number, conformance to guidelines

The funds requested are essential and justified by the importance and scientific potential of the research, investigator time and proposed involvement related to management of the research. The proposal demonstrates value for money in terms of the resources requested as it will create valuable research infrastructure as well as opportunities for fellows and junior investigators to conduct translational research in the realm of FH awareness, detection and control.

Overall Assessment

Score 1-6

1 - Poor	2 - Good	3 - High	4 - Very High	✔ 5 - Excellent	6 - Exceptional

Medical Research Council
2nd Floor David Phillips Building, Polaris House, North Star Avenue, Swindon,
United Kingdom SN2 1ET
Telephone +44 (0) 1793 416200
Web http://www.mrc.ac.uk/

COMPLIANCE WITH THE DATA PROTECTION ACT 1998

In accordance with the Data Protection Act 1998, the personal data provided on this form will be processed by MRC, and may be held on computerised database and/or manual files. Further details may be found in the **guidance notes**

Research Grant
Peer Review

MRC Reference: MR/T017384/1

Document Status: With Council

UK-Malaysia Health Research Partnership

2019

Applicant Details

Applicant	Professor Nadeem Qureshi	Organisation	University of Nottingham

Title of Research Project

Reducing Premature Coronary Artery Disease in Malaysia by early identification of Familial Hypercholesterolaemia

Review Information

Response Due Date	12/08/2019	Reviewer Reference:	083603263

Research Quality

Research Quality: Please comment on the importance and competitiveness of the proposed research, including:

(1) strength of medical or scientific case (2) level of innovation, and whether this is likely to lead to significant new understanding (3) management strategy proposed, including equitable access to any shared resources (4) feasibility of experimental plans, statistics, methodology and design, including provision of sample size calculations, strategies to avoid bias, and preliminary data where appropriate (5) how well risks have been identified, and will be mitigated.

This proposal seeks to introduce a method of FH identification into Malaysian healthcare by comparing 3 identification methods and evaluating screening performance and acceptability. The three methods include FAMCAT (regarded as a screening test), Simon Broome and the Dutch criteria (regarded as diagnostic tests) and focus on testing adults in a primary care setting. There are problems with the proposal.

1. The strength of the medical and scientific case in limited
2. The level of innovation is low and is unlikely to lead to significant new understanding
3. The identification strategy and methodology has a fundamental conceptual flaw (a circularity where screening and diagnosis are defined by the same factors)
4. The feasibility of introducing the plan is reasonable but the statistical approach to assessing screening performance, methodology and strategies to avoid circular bias are a problem.
5. No measures appear to be in place to mitigate these problems/risks

The scientific basis for this assessment is set out below.

1. There is a fundamental conceptual flaw in the proposal that the disorder (FH) is defined by its screening test. This creates a circularity that makes it impossible to assess screening performance accurately. For example, if a cholesterol measurement is used in screening and then again in diagnosis, it will overestimate screening performance.
2. It is therefore invalid to use measures such as area under the curve, detection rate (sensitivity) or false positive rate (1-specificity). In the extreme situation, using the same variables in screening as in the definition of FH will create the illusion of perfect screening performance.
3. Each proposed method (FAMCAT, SB or Dutch) uses a combination of variables in screening that include LDL cholesterol, Total cholesterol, family history , history of myocardial infarction and an FH mutation. The definition of the disorder (FH) also includes these variables (so the circularity is always there), but the problems are then compounded because multiple definitions of the disorder are used depending on whether the diagnosis is definite, possible or probable.
4. Any screening programme should be based on a policy that leads to a clear diagnosis, not a graduated one. The notion of probable and possible FH is largely historical, when the use of genomics was unavailable. Now one can be more definite by defining FH as a high cholesterol (the causal intermediate phenotype) supported by an FH mutation and this should replace the need for uncertain probable and possible categories.
5. Notwithstanding these problems, it is also strange that the applicants have chosen to compare a screening Method (FAMCAT) with diagnostic Methods (Simon Broom and Dutch criteria). It would be more usual to see different screening methods (eg FAMCAT and some of the other screening methods available eg. SEARCH), compared against a single diagnostic test/definition.
6. There is evidence that cholesterol measurement in adults is not as good at identifying individuals with FH (whether defined clinically or by DNA) as testing in children; it is less than half as discriminatory. It is therefore a backwards step to seek to introduce an FH identification method that centres in testing adults rather than children (and then the parents of affected children, one of whom will be positive).
7. Use of Area under the Curve has been shown to be an unsatisfactory measure of screening performance (even if the circular limitation above was not a major issue) because it combines the two important variables (detection rate and false positive rate) into one - when what is important is each individually and the trade off between the two. It is possible that the same area under the curve could be associated with quite different detection rates and false positive rates, if the curve is skewed, and this will give a false impression of screening performance.

Research Environment and People

Please comment on the suitability of the investigator group and the environment where the proposed research will take place, including (1) track record(s) of the individuals in their field(s) and whether they are best-placed to deliver the proposed research (2) level of commitment of host research organisation to supporting the proposed research (3) whether appropriate facilities will be available to the researchers

1. Track record of UK investigators is good and they have they published findings showing that FAMCAT (the screening test) is better than Simon Broome or the Dutch criteria (the diagnostic tests) in identifying individuals with FH. Whilst this is surprising given that an FH mutation is used in the diagnostic tests but not in screening, if true, it suggests that the screening test should replace the diagnostic test. If the results are correct it is unclear why the results should be markedly different in other countries like Malaysia.
2. Not clear what role the host institutions will play in supporting the proposed research. How likely is it that the primary care data sets in Malaysia will be complete? In the UK there are many gaps in data which require imputation. How much imputation will be needed in Malaysia and to what extent does this averaging diminish the reliability of the results? How will the host institutions help with this?
3. It is likely that facilities will be available, although hard to comment confidently.

Impact

Please comment on the potential economic and societal impact of the proposed research, including (1) identification of realistic potential improvements to human or population health (2) contribution to relieving disease/disability burden and/or improving quality of life (3) identification of potential impacts of research and plans to deliver these (in the Pathways to Impact statement)

1. It is likely that any adult screening method will identify some cases of FH but there will also be considerable mis-classification because testing in adults in far less accurate than testing in childhood. Screening adults is also a problem because FH causes fatal and non-fatal ischaemic heart disease events in the young so many such events will be missed by restricting testing in adults.
2. To the extent that some adults are identified this is a benefit but the overall societal impact is likely to be limited by the late age of screening.
3. Any research on FH in a country that has little focus on FH is likely to increase the profile of the condition and awareness and to this extent is a benefit/impact.

Ethics

Please comment on any ethical and/or research governance issues, including (1) whether proposed research is ethically acceptable (2) any ethical issues that need separate consideration (3) appropriateness of ethical review and research governance arrangements (4) any potential adverse consequences for humans, animals or the environment and whether these risks have been addressed satisfactorily in the proposal

No ethical concerns

Data Management Plan

Please assess whether the data management plan indicates whether the applicants have (or are likely to have) a sound plan for managing the research data funded through the award, taking into account (1) the types, scale and complexity of data being (or to be) managed; (2) the likely long-term value for further research including by sharing data; and (3) the anticipated information security and ethics requirements.

1. The primary applicant has experience in managing data of this kind and on a scale of this kind
2. Limited long-term value of data
3. No foreseen security and ethics requirements beyond the usual requirements of data Protection and GDPR (to the extent that this applies in Malaysia)

Resources Requested

Please comment on (1) whether funds requested are essential and justified by the importance and scientific potential of the research (2) investigator time and proposed involvement related to management of the research (3) whether the proposal demonstrates value for money in terms of the resources requested (4) whether any animal use is fully justified in terms of need, species, number, conformance to guidelines

1. Difficult to comment on funding given the scientific concerns raised above, but an argument that such funds could more usefully be used implementing a child-focussed screening programme, which will also identify the parent at a younger age, before the onset of clinical disease (IHD).
2. Investigator time seems somewhat low for a project that will require considerable oversight.
3. Introducing screening for FH to Malaysia would be a valuable exercise and worth investment but this approach is not the

most effective approach available<br>4. N/A

Overall Assessment

Score 1-6

✔ 1 - Poor	2 - Good	3 - High	4 - Very High	5 - Excellent	6 - Exceptional

Medical Research Council
2nd Floor David Phillips Building, Polaris House, North Star Avenue, Swindon,
United Kingdom SN2 1ET
Telephone +44 (0) 1793 416200
Web http://www.mrc.ac.uk/

COMPLIANCE WITH THE DATA PROTECTION ACT 1998
In accordance with the Data Protection Act 1998, the personal data provided on this form will be processed by MRC, and may be held on computerised database and/or manual files. Further details may be found in the **guidance notes**

Research Grant
Peer Review

MRC Reference: MR/T017384/1

Document Status: With Council

UK-Malaysia Health Research Partnership

2019

Applicant Details

Applicant	Professor Nadeem Qureshi	Organisation	University of Nottingham

Title of Research Project

Reducing Premature Coronary Artery Disease in Malaysia by early identification of Familial Hypercholesterolaemia

Review Information

Response Due Date	06/08/2019	Reviewer Reference:	122878178

Research Quality

Research Quality: Please comment on the importance and competitiveness of the proposed research, including:

(1) strength of medical or scientific case (2) level of innovation, and whether this is likely to lead to significant new understanding (3) management strategy proposed, including equitable access to any shared resources (4) feasibility of experimental plans, statistics, methodology and design, including provision of sample size calculations, strategies to avoid bias, and preliminary data where appropriate (5) how well risks have been identified, and will be mitigated.

(1) strength of medical or scientific case :
The key idea, the testing of FAMCAT in another setting is an important question to answer in order to provide evidence for systematic efforts to detect and manage FH patients and families, with research activities supporting the development of clinical pathways and process with imput from users.
(2) level of innovation:
The innovation element is essentially the validation of the tool and in identifying user's experiences, concerns and expectations
(3) management strategy proposed
Plans well structured.
(4) feasibility of experimental plans, statistics, methodology and design
In general, the plans are clear and feasible and adequately funded.

One concern is in the selection of the clinics to be included, as there is no details on the methods (eg random or purposively sampled) and little detail on the analysis plan. For example, while the main outcome is explicitly mentioned, little detail on other variables to be collected is not presented. Some of these can be particularly useful for infomring the

development of the guides and pathways.

(5) how well risks have been identified, The main risk, data security is well described and data management standards are high.

Research Environment and People

Please comment on the suitability of the investigator group and the environment where the proposed research will take place, including (1) track record(s) of the individuals in their field(s) and whether they are best-placed to deliver the proposed research (2) level of commitment of host research organisation to supporting the proposed research (3) whether appropriate facilities will be available to the researchers

The research team is experienced nationally and internationally, wit a good mix of expertise and experience.
The researchers are supported by their host organization, as well they have good access to primary care settings to conduct the research.

Impact

Please comment on the potential economic and societal impact of the proposed research, including (1) identification of realistic potential improvements to human or population health (2) contribution to relieving disease/disability burden and/or improving quality of life (3) identification of potential impacts of research and plans to deliver these (in the Pathways to Impact statement)

(1) identification of realistic potential improvements to human or population health:
the streamlining of pathways and supporting tools to identify FH cases are likely to result in benefit for the country health, will provide useful information for local guidelines development and adaptation.

(2) contribution to relieving disease/disability burden and/or improving quality of life:
The proposed engagement with index cases is an interesting idea, as it might provide useful information for guidelines development.

(3) identification of potential impacts of research and plans to deliver these (in the Pathways to Impact statement).
The benefits for the academic community are clearly described, specially the tools and datasets that the study will generate.
The communication plan with health authorities is a potential excellent way of increasing the chhances for the research to influence processes related FH detection in Malaysia.
There is little detail on how and who will be engaged in the co-production elements, nor specific elements in the budget have been identified for these activities.

Ethics

Please comment on any ethical and/or research governance issues, including (1) whether proposed research is ethically acceptable (2) any ethical issues that need separate consideration (3) appropriateness of ethical review and research governance arrangements (4) any potential adverse consequences for humans, animals or the environment and whether these risks have been addressed satisfactorily in the proposal

No issues with ethics plan.

Data Management Plan

Please assess whether the data management plan indicates whether the applicants have (or are likely to have) a sound plan for managing the research data funded through the award, taking into account (1) the types, scale and complexity of data being (or to be) managed; (2) the likely long-term value for further research including by sharing data; and (3) the anticipated information security and ethics requirements.

Well explained, and good idea of a two tiers access to data and tools based on country income classification.

Resources Requested

Please comment on (1) whether funds requested are essential and justified by the importance and scientific potential of the research (2) investigator time and proposed involvement related to management of the research (3) whether the proposal demonstrates value for money in terms of the resources requested (4) whether any animal use is fully justified in terms of need, species, number, conformance to guidelines

Budget reasonable for the proposed activities, although several impact activities not funded in the central budget.

Overall Assessment

Score 1-6

1 - Poor	2 - Good	3 - High	✔ 4 - Very High	5 - Excellent	6 - Exceptional

Medical Research Council
2nd Floor David Phillips Building, Polaris House, North Star Avenue, Swindon,
United Kingdom SN2 1ET
Telephone +44 (0) 1793 416200
Web http://www.mrc.ac.uk/

COMPLIANCE WITH THE DATA PROTECTION ACT 1998

In accordance with the Data Protection Act 1998, the personal data provided on this form will be processed by MRC, and may be held on computerised database and/or manual files. Further details may be found in the **guidance notes**

Research Grant Peer Review

MRC Reference: MR/T017384/1

Document Status: With Council

UK-Malaysia Health Research Partnership

2019

Applicant Details

Applicant	Professor Nadeem Qureshi	Organisation	University of Nottingham

Title of Research Project

Reducing Premature Coronary Artery Disease in Malaysia by early identification of Familial Hypercholesterolaemia

Review Information

Response Due Date	29/07/2019	Reviewer Reference:	124471833

Research Quality

Research Quality: Please comment on the importance and competitiveness of the proposed research, including:

(1) strength of medical or scientific case (2) level of innovation, and whether this is likely to lead to significant new understanding (3) management strategy proposed, including equitable access to any shared resources (4) feasibility of experimental plans, statistics, methodology and design, including provision of sample size calculations, strategies to avoid bias, and preliminary data where appropriate (5) how well risks have been identified, and will be mitigated.

The applicant claimed that the proposed project will indicate an acceptable and effective approach to identify FH in Malaysian primary care and a clearer picture of FH-causing genetic mutations that are found in the Malaysian population leading to better tailored genetic tests. The interviews with suspected FH, primary care doctors and nurses will be able to provide advice to the Ministry of Health and policymakers on how to introduce FH identification in primary care and clinical pathway once tested. This includes advising on guidelines, the training need of health professionals and public health campaigns. Generally, this proposed study will benefit Malaysian society through reduction in premature CAD, especially in those from the low and middle income groups. Applicant did not mentioned clearly how findings worldwide different from the proposed study since there are many similar studies worldwide.

Applicant did not clearly show how sample size calculation based on multiethnics and also indigenous people. Applicant proposed 6 urban and 6 suburban clinics in the sampling for Workstream 1. Malaysia comprises of 13 states, not sure if 6 urban and 6 suburban clinics selected represent Malaysia population well as more indigenous people with different ethnics in Sabah and Sarawak compare to peninsular Malaysia.

Applicant only proposed 310 respondents for Work Stream 2. What about control group?

The proposed study lacks of in-depth statistical analyses, appropriate project risks and detail of the mitigation.

Research Environment and People

Please comment on the suitability of the investigator group and the environment where the proposed research will take place, including (1) track record(s) of the individuals in their field(s) and whether they are best-placed to deliver the proposed research (2) level of commitment of host research organisation to supporting the proposed research (3) whether appropriate facilities will be available to the researchers

All the investigators involve in the study are approprite and demonstrated substantial expertise related to the proposed study.

The proposed study covers 6 urban and 6 suburban clinics in the sampling for Workstream 1. Malaysia comprises of 13 states, not sure if 6 urban and 6 suburban clinics selected represent Malaysia population well as more indigenous people with different ethnics in Sabah and Sarawak compare to peninsular Malaysia.

Impact

Please comment on the potential economic and societal impact of the proposed research, including (1) identification of realistic potential improvements to human or population health (2) contribution to relieving disease/disability burden and/or improving quality of life (3) identification of potential impacts of research and plans to deliver these (in the Pathways to Impact statement)

The direct impact of the study includes:
1. identification of the most appropriate primary care FH case identification tool to opportunistically identify suspected FH patients.
2. Genetic mutation profile of individuals with suspected FH in the general population.
3. Identification of new variant mutations in the 7 FH candidate genes to support development of genetic mutation testing for FH that is relevant to the Malaysian population.
4. An insight into patients and practitioners opinions of the intervention through qualitative research.

Applicant did not clearly mentioned how the findings benefits various ethnics which includes huge portion of indigenous people in Malaysia.

Ethics

Please comment on any ethical and/or research governance issues, including (1) whether proposed research is ethically acceptable (2) any ethical issues that need separate consideration (3) appropriateness of ethical review and research governance arrangements (4) any potential adverse consequences for humans, animals or the environment and whether these risks have been addressed satisfactorily in the proposal

Applicant aware with the appropriate ethicby The Regional Multicentre Research Ethics Committee (MREC) or Local Research Ethics Committee (LREC)s required for the proposed study.
The study have not given any approval by The Regional Multicentre Research Ethics Committee (MREC) or Local Research Ethics Committee (LREC) currently.

Data Management Plan

Please assess whether the data management plan indicates whether the applicants have (or are likely to have) a sound plan for managing the research data funded through the award, taking into account (1) the types, scale and complexity of data being (or to be) managed; (2) the likely long-term value for further research including by sharing data; and (3) the anticipated information security and ethics requirements.

The proposed study includes appropriate information on the following:
1. Type of data
2. Data management
3. Data security and confidential
4. Data sharing and access

Resources Requested

Please comment on (1) whether funds requested are essential and justified by the importance and scientific potential of the research (2) investigator time and proposed involvement related to management of the research (3) whether the proposal demonstrates value for money in terms of the resources requested (4) whether any animal use is fully justified in terms of need, species, number, conformance to guidelines

The funds requested essential for the work and justified by the importance and scientific potential of the research.
Applicant stated sufficient time commitment to the work.

The proposal demonstrate limited value for money in terms of the resources requested because Malaysia is a multi-ethnic country and the proposed study did not well address the indigenous group.

Overall Assessment

Score 1-6

1 - Poor	✔ 2 - Good	3 - High	4 - Very High	5 - Excellent	6 - Exceptional

Medical Research Council
2nd Floor David Phillips Building,
Polaris House, North Star Avenue,
Swindon,
United Kingdom SN2 1ET
Telephone +44 (0) 1793 416200
Web http://www.mrc.ac.uk/

COMPLIANCE WITH THE DATA PROTECTION ACT 1998
In accordance with the Data Protection Act 1998, the personal data provided on this form will be processed by MRC, and may be held on computerised database and/or manual files. Further details may be found in the **guidance notes**

Research Grant
Peer Review

MRC Reference: MR/T017384/1

Document Status: **With Council**

UK-Malaysia Health Partnership 2019 Call

Peer Review

Applicant Details

Applicant	Professor Nadeem Qureshi	Organisation	University of Nottingham

Title of Research Project

Reducing Premature Coronary Artery Disease in Malaysia by early identification of Familial Hypercholesterolaemia

Research Quality

Please comment on the importance and competitiveness of the proposed research, including:

(1) strength of medical or scientific case (2) level of innovation, and whether this is likely to lead to significant new understanding (3) management strategy proposed, including equitable access to any shared resources (4) feasibility of experimental plans, statistics, methodology and design, including provision of sample size calculations, strategies to avoid bias, and preliminary data where appropriate (5) how well risks have been identified, and will be mitigated.

This proposal has both practical application and will contribute significantly to the canon of scientific knowledge specifically about an important genetic disorder and generally as a model for the identification of genetic variants with disease-producing potential. Effective treatment is available for the familial hypercholesterolaemia (FH) clinical phenotype with cheaply available statin drugs and ezetimibe. The design is robust and

the implementation of this proposal highly feasible. It has more than adequate statistical power to answer the research questions posed.

Research Environment and People

Please comment on the suitability of the investigator group and the environment where the proposed research will take place, including (1) track record(s) of the individuals in their field(s) and whether they are best-placed to deliver the proposed research (2) level of commitment of host research organisation to supporting the proposed research (3) whether appropriate facilities will be available to the researchers

Excellent facilities for this research appear to be available in all the sites involved. The investigators are amongst the leading exponents of this type of research internationally and clearly have the necessary expertise, enthusiasm and drive to complete the project successfully.

Impact

Please comment on the potential economic and societal impact of the proposed research, including (1) identification of realistic potential improvements to human or population health (2) contribution to relieving disease/disability burden and/or improving quality of life (3) identification of potential impacts of research and plans to deliver these (in the Pathways to Impact statement)

Heterozygous FH is the commonest genetic disorder of sufficient penetrance to cause severe disease in most populations other than those of pure African descent. Generally in Europe and populations of European descent it is expressed in 1 in 250-500 people producing premature coronary heart disease due to hypercholesterolaemia in the majority. Its effects can be considerably ameliorated, if it is identified. In some populations it may have a higher prevalence. Previously this has been reported in South Africa, the Lebanon and French Canadians. The discovery that a high prevalence in Malaysia may also be the case is of great interest. Previously it has been reported that in Chinese families, in which FH-causing genes are running, on migration to Canada a more severe phenotype develops in affected members. The combination of the major dietary change occurring in Malaysia with high prevalence of FH presents a major public health challenge and a most interesting research opportunity.

Ethics

Please comment on any ethical and/or research governance issues, including (1) whether proposed research is ethically acceptable (2) any ethical issues that need separate consideration (3) appropriateness of ethical review and research governance arrangements (4) any potential adverse consequences for humans, animals or the environment and whether these risks have been addressed satisfactorily in the proposal

I see no ethical objections to this protocol

Data Management Plan

Please assess whether the data management plan, indicates whether the applicants have (or are likely to have) a sound plan for managing the research data funded through the award, taking account the (1) the types, scale and complexity of data being (or to be) managed; (2) the likely long-term value for further research including by sharing data; and (3) the anticipated information security and ethics requirements.

They have an excellent, world class track record in handling the type of data which will be generated. They are committed to dissemination by publication of their findings and by other means leading to their translation into clinical practice.

Resources Requested

Please comment on (1) whether funds requested are essential and justified by the importance and scientific potential of the research (2) investigator time and proposed involvement related to management of the research (3) whether the proposal demonstrates value for money in terms of the resources requested (4) whether any animal use is fully justified in terms of need, species, number, conformance to guidelines

The resources requested are adequate and by no means inflated.

Overall Assessment

Score 1-6

☐ 1 - Poor	☐ 2 - Good	☐ 3 - High	☐ 4 - Very High	☐ 5 - Excellent	**X**☐ 6 - Exceptional

Medical Research Council
2nd Floor David Phillips Building, Polaris House, North Star Avenue, Swindon,
United Kingdom SN2 1ET
Telephone +44 (0) 1793 416200
Web http://www.mrc.ac.uk/

COMPLIANCE WITH THE DATA PROTECTION ACT 1998
In accordance with the Data Protection Act 1998, the personal data provided on this form will be processed by MRC, and may be held on computerised database and/or manual files. Further details may be found in the **guidance notes**

Research Grant Peer Review

MRC Reference: MR/T017384/1

Document Status: With Council

UK-Malaysia Health Research Partnership

2019

Applicant Details

Applicant	Professor Nadeem Qureshi	Organisation	University of Nottingham

Title of Research Project

Reducing Premature Coronary Artery Disease in Malaysia by early identification of Familial Hypercholesterolaemia

Review Information

Response Due Date	29/07/2019	Reviewer Reference:	189609547

Research Quality

Research Quality: Please comment on the importance and competitiveness of the proposed research, including:

(1) strength of medical or scientific case (2) level of innovation, and whether this is likely to lead to significant new understanding (3) management strategy proposed, including equitable access to any shared resources (4) feasibility of experimental plans, statistics, methodology and design, including provision of sample size calculations, strategies to avoid bias, and preliminary data where appropriate (5) how well risks have been identified, and will be mitigated.

The optimal approach to ascertainment of cases of familial hypercholesterolaemia remains uncertain, and there is a clear unmet need. People with FH have higher risks of CHD and this can be reduced by statin treatment. Too often FH is diagnosed at the time of a first MI illustrating the missed opportunity for primary prevention.

In Malaysia as in all other countries the reasons for this are:

1) Only a minority of people with mutations leading to FH display cutaneous features (xanthelesma and xanthoma)
2) Blood LDL-C values do not adequately distinguish people with monogenic FH (for whom cascade screening in families would be worthwhile) from those with polygenic hypercholesterolaemia (for whom cascade screening would not be worthwhile)
3) Many families with FH have mutations in the LDL-receptor but these vary among FH families precluding mutation testing as a low cost high throughput means of diagnosis and making targeted sequencing of LDLR (APOB and PCSK9 genes) the optimal approach to diagnosing monogenic FH
4) Targeted sequencing is prohibitively expensive as a primary route to population screening

Therefore the motivating aims of the project are sound.

The applicants argue that Malaysia has a particular problem because monogenic FH may be more prevalent than elsewhere (1 per 100, c.f. 1 in 250 in the UK) and rates of premature CHD are high. However, no comparative figures are provided to help judge the strength of this assertion.

The applicants core proposal is that the new FAMCAT screening tool, developed by the lead applicant in the UK, has a superior performance to the Simon-Broome (S-B) and Dutch Lipid Clinic Criteria (DLCC) for screening for FH cases. All three tools / criteria are based on some combination of routinely recorded features in the history, clinical examination +/- blood cholesterol measurement. The superiority of the FAMCAT tool is claimed based on two very large studies using UK primary care records (one inception, one validation) in which FAMCAT displayed a higher discrimination for FH than S-B or DLCC in the same dataset. However, a critical limitation of both studies is that it is uncertain if patients with the diagnosis of FH in the primary care record were mutation positive or not. Since it is known that people with a high burden of common LDL-C alleles can phenocopy monogenic FH, the performance of any of the criteria or tools for detecting monogenic FH remains uncertain.

The proposal, commendably seeks to compare the performance of FAMCAT bs S-B and DLCC in detecting FH cases, however, the design of the study (Aims 1 and 2; the main aims) has some limitations:

1) Participants will be recruited from those attending primary care clinics for other reasons. It seems unlikely that this sampling frame would yield an estimate of the population incidence of FH since clinic attenders may not be representative of the population at large
2) the study hypothesis seems to be that FAMCAT will outperform S-B and DLCC, and therefore sequencing of the known FH genes will be limited to those individuals that screen positive based on FAMCAT. However, if S-B or DLCC prove to outperform FAMCAT some individuals who screen positive for S-B or DLCC but not FAMCAT would miss being sequenced. A better design would have been to sequence all those who screened positive with any of the tools.
3) There seem to be no plans to sequence even a random sample of those who screen negative for FAMCAT. This is important as it is known that some mutation carriers do not display a phenotype.

Aims 3 and 4 are around qualitative aspects of the work.

Research Environment and People

Please comment on the suitability of the investigator group and the environment where the proposed research will take place, including (1) track record(s) of the individuals in their field(s) and whether they are best-placed to deliver the proposed research (2) level of commitment of host research organisation to supporting the proposed research (3) whether appropriate facilities will be available to the researchers

The groups in Nottingham and Malaysia have a very strong track record of research in this area including national policy roles.

Impact

Please comment on the potential economic and societal impact of the proposed research, including (1) identification of realistic potential improvements to human or population health (2) contribution to relieving disease/disability burden and/or improving quality of life (3) identification of potential impacts of research and plans to deliver these (in the Pathways to Impact statement)

A cost-efficient and acceptable approach to FH screening would be very valuable if optimally designed and hold the prospect of substantially reducing the burden of CHD.

Ethics

Please comment on any ethical and/or research governance issues, including (1) whether proposed research is ethically acceptable (2) any ethical issues that need separate consideration (3) appropriateness of ethical review and research governance arrangements (4) any potential adverse consequences for humans, animals or the environment and whether these risks have been addressed satisfactorily in the proposal

No concerns

Data Management Plan

Please assess whether the data management plan indicates whether the applicants have (or are likely to have) a sound plan for managing the research data funded through the award, taking into account (1) the types, scale and complexity of data being (or to be) managed; (2) the likely long-term value for further research including by sharing data; and (3) the anticipated information security and ethics requirements.

No concerns

Resources Requested

Please comment on (1) whether funds requested are essential and justified by the importance and scientific potential of the research (2) investigator time and proposed involvement related to management of the research (3) whether the proposal demonstrates value for money in terms of the resources requested (4) whether any animal use is fully justified in terms of need, species, number, conformance to guidelines

No concerns

Overall Assessment

Score 1-6

1 - Poor	2 - Good	3 - High	✔ 4 - Very High	5 - Excellent	6 - Exceptional

Response to Reviewers for Grant Ref: MR/T017384/1

Reducing Premature Coronary Artery Disease in Malaysia by early identification of Familial Hypercholesterolaemia (FH)

We appreciate the six reviewers' comments and provide a summary of our response, and a specific response to each reviewer below.

SUMMARY: We underline the proposed study is a head to head comparison of different approaches to identify possible cases of FH in primary care. This uses internationally recognised standard criteria for adult case finding: Dutch Lipid Clinic Criteria (DLCC), Simon-Broome (SB) and a new approach (FAMCAT). We note that DLCC and SB criteria may be used to identify possible cases in non-specialist settings, such as primary care, [1] and in specialist settings to clinically diagnose FH.[2] In the proposed study, the performance of each clinical approach will be clearly assessed against genetic testing, rather than specialist clinical diagnosis or FH documented in primary care records. Following extensive feasibility work, this study is designed for current Malaysian primary care, by specifically recruiting patients who have already had routine cholesterol tests in primary care. Currently, children do not have these tests. The study is not introducing new systematic public health screening. Within the resources available, including primary and specialist care services, the study focuses on the diverse multi-ethnic communities of the greater Kuala Lumpur/Putrajaya area. The main aim is identifying possible index cases of FH, hence the impact of cascade testing to relatives is not a specific objective.

Reviewer_143010184: Score 6. We thank the reviewer for recognising the proposal is feasible, and our ability to deliver the study. The observation that FH may have a more severe phenotype in Chinese and related ancestry also further supports the benefits of this research.

Reviewer_189609547: Score 4. We thank the reviewer for noting there is an unmet need for adult case finding of FH, and the team's track record in relevant research and health policy.

Specific queries on prevalence of FH and premature coronary artery disease (CAD) in Malaysia: The 1 in 100 prevalence of FH is based on voluntary community screening and compared to other countries in the cited reference.[3] We note 23.8% of CAD is among those under 50 years, which is younger than other neighbouring countries.[4]

Evaluating SB, DLCC & FAMCAT & sampling frame: As in the summary (above), we note: (i) The primary objective and the sampling frame seek to compare case finding strategies. (ii) Based on big data analysis in the UK and related implementation [5], we hypothesise that FAMCAT will have a better detection rate than SB & DLCC in Malaysia. However this can only be confirmed by genetic testing, as proposed. All patients identified with possible FH by SB, DLCC or FAMCAT approaches will be offered genetic testing/sequencing. This includes patients who may be SB/DLCC positive but FAMCAT negative.

Reviewer_083603263: Score 1. We note the reviewer regards our approach as feasible and the strength of the study team. We appreciate the reviewer's enthusiasm for childhood FH screening. However, this does not negate the benefits of improving adult case finding that might be achieved in current primary care, as discussed in the Summary above. Overall the reviewer's comments indicate some misunderstanding (for example about the difference between case finding and screening) or are not supported by evidence. Below we respond to this reviewer's criticism in each section, with evidence to support and justify our proposed approach:

Research Quality: Points 1,3-6 (*circularity, concept, clinical criteria*). The reviewer suggests that the diagnosis is made by the same criteria used to identify patients who have a possible (high pre-test probability) of FH (FAMCAT, DLCC, SB). This is entirely incorrect – In Work stream 1, we clearly state that the diagnosis is made by genetic testing (in case for support (page 4). In fact the primary outcome is the detection rate of genetically-confirmed FH.

The proposed approach of using clinical criteria in primary care to select for referral to specialists and genetic testing is that recommended by numerous international guideline committees including, UK NICE guidelines, EU ESC/EAS guidelines, US guidelines, Japanese guidelines [6-9] Hence our approach is very clearly policy-based, with SB and DLCC the most commonly used clinical criteria internationally. For instance, in the UK, NICE guidelines (CG71 – section 1.1) make a distinction between "case-finding/screening" and "diagnosis", and diagnosis is made by referral of those who meet possible or definite SB clinical criteria and/or DLCC score above 4 for confirmatory genetic testing (recommendation 1.1.6).[6] Indeed, contrary to this reviewer's comment, another Reviewer 189609547, highlights that "Targeted (genetic) sequencing is prohibitively expensive as a primary route to population screening."

Points 2, 7: To re-iterate, the proposed primary outcome is defined by genetic testing and not the clinical criteria which this reviewer has suggested. Hence, metrics such as AUC, detection rate, sensitivity, specificity will all apply here. Numerous studies internationally, both published and ongoing, have adopted this approach.[1,2,10] We agree AUC has limitations, and have proposed to assess other metrics of performance including overall detection rate, net reclassification index, C-index, and calibration.

Point 5: As per standard observational study design, FAMCAT is compared to standard clinical criteria (DLCC & SB) as described above. The reviewer suggested the SEARCH tool, but this is actually an enhanced electronic version of the DLCC.

Point 6: As indicated above, the proposed research uses international evidence-based policy recommendations on adult case finding [6-9]. Childhood FH screening is still not recommended and is not feasible within current Malaysian primary care infrastructure and referral pathways.[11]

Research Environment: We anticipate results will be different in Malaysia, given that the clinical criteria (FAMCAT, SB, DLCC) are derived from Western populations. The genetic profiles are also likely to be different, hence we are investigating genetic profile in Workstream 2. Moreover, in our recent external validation of FAMCAT there were significant differences in FH detection in different UK ethnic groups.[12] The host institution in Malaysia (UTiM) is the national centre of excellence in FH and has state-of-art genetic testing facilities. The feasibility of collecting clinical criteria, related medical and demographic data in Malaysian primary care clinics has been confirmed in our recent pilot, with 405 patients assessed over 20 working days, and nearly 90% data completion rate, which is far better than data quality from UK primary care records. In the case of missing data, as laid out in our statistical analysis plan, standard imputation techniques (multiple imputation using chained equations) will be used. [13]

Impact, Data Management & Resources: We thank the reviewer for acknowledging our experience in managing this kind of project, on this scale. The reviewers comments on *adult case finding* are contrary to international guideline recommendations that recognise the evidence of long-term benefit with this approach, including preventing premature CAD and identifying other affected relatives including children and young adults.[14,15] The reviewer suggests there is limited long-term value for the study data. We disagree. We would strongly highlight that this will be the first study to derive a cohort of patients identified with FH in Malaysian primary care with full genetic profiling. Most FH cohorts are derived from Western populations and clinical criteria are skewed towards these populations. Finally, there is still only limited evidence that resources should be shifted from adult case finding to childhood screening, and this is not currently applicable for Malaysian primary care.

Reviewer_079046588: Score 5. We thank this reviewer for recognising the study as innovative with a strong scientific and medical basis, and noting that risk/hurdles have been addressed. Considering limitations, we would note: (1) the primary objective of study is not cascading testing to family members; (2) Variant frequencies will be obtained from the Malaysian 1000 Gene database. (3) The data on cases is being captured in a bespoke database (FH CatScreen), not

directly from electronic health records. (4) We agree secondary cases should be excluded, and this is incorporated in the FH CatScreen database.

Reviewer_124471833: Score 2. We thank the reviewer for recognising strength of the team. In response to his/her query, thus far, we have not identified a comparable study in primary care.[1] This study will recruit patients with Malay, Chinese and Indian ancestry but due to limited resources and practical issues of recruitment, we are unable to extend recruitment nationally to other ethnic groups. However once this study is completed, we agree the natural next step is a national implementation/clinical utility study. From Workstream 1, we will recruit 310 unrelated index FH patients. In Workstream 2, the genetic profile of this cohort will still be informative. If future funding is available, we will include 310 unaffected family members as controls. We would like to direct this reviewer to the Annex 1 provided for the statistical analyses.

Reviewer_122878178: Score 4. We thank the reviewer for their positive review, in particular acknowledging we have addressed the main risk mitigation related to data management, and our innovative impact strategy with health authorities.

As indicated in Annex 1, for this observational study we are purposefully sampling primary clinics with diverse populations. Statistical analysis is summarised in Annex 1 and workstreams. The analyses will incorporate demographic data and data on diagnostic variables for FAMCAT, SB and DLCC criteria. Qualitative data analysis will inform guideline and pathway development. The co-production elements will also be informed by parallel qualitative research. Our current proposal is to include clinical pathway/guideline development and public health campaigns, through engagement with the Ministry of Health and other key primary and specialist care stakeholders.

REFERENCES

(1) Qureshi N, et al. Strategies for identifying familial hypercholesterolaemia in non-specialist clinical settings. *Cochrane Database of Systematic Reviews* 2018, Issue 3. Art. No.: CD012985.
(2) Haralambos K et al. Clinical experience of scoring criteria for Familial Hypercholesterolaemia genetic testing in Wales. *Atherosclerosis* 240 (2015) 190e196
(3) Vallejo-Vaz, A., et al, Overview of the current status of familial hypercholesterolaemia care in over 60 countries - The EAS Familial Hypercholesterolaemia Studies Collaboration (FHSC). *Atherosclerosis*, 2018. 277: p. 234-255.
(4) National Heart Association and Ministry of Health Malaysia. Annual Report of the NCVD-ACS Registry 2014-2015. https://www.malaysianheart.org/files/5ae6b04 77ed84.pdf
(5) PRIMIS Familial Hypercholesterol QI tool. https://www.nottingham.ac.uk/primis/tools/qi-tools/familial-hypercholesterolaemia.aspx
(6) National Institute for Health and Care Excellence. *Identification and management of familial hypercholesterolaemia.* NICE clinical guideline 71. London, 2008 [Updated 2017].
(7) Reiner Ž, et al. ESC/EAS Guidelines for the management of dyslipidaemias: The Task Force for the management of dyslipidaemias of the European Society of Cardiology (ESC) and European Atherosclerosis Society (EAS). *Euro Heart Journal* 2011; 32(14): 1769-818.
(8) Grundy SM, et al. Guideline on the Management of Blood Cholesterol: Executive Summary: A Report of the American College of Cardiology Task Force on Clinical Practice Guidelines. *Journal of the American College of Cardiology* 2019; 73(24): 3168-209.
(9) Harada-Shiba M, et al. Guidelines for Diagnosis and Treatment of Familial Hypercholesterolemia 2017. *J Atheroscler Thromb* 2018; 25(8): 751-70. Japanese guidelines
(10) Arnold-Reed DE, et al. Detection and management of familial hypercholesterolaemia in primary care in Australia: protocol for a pragmatic cluster intervention study with pre-post intervention comparisons. *BMJ Open* 2017; 7(10): e017539.
(11) UK National Screening Committee. *Screening for familial hypercholesterolaemia in childhood.* https://legacyscreening.phe.org.uk/familialhypercholesterolaemia-child
(12) Weng S, et al.. Detection of Familial Hypercholesterolaemia: external validation of the FAMCAT clinical case-finding algorithm to identify patients in primary care. *Lancet Public Health* 2019; **4**(5): e256-e64)
(13) Royston P. Multiple imputation of missing values: Update of ice. *The Stata Journal* 2005; **5**(4): 527-36.)
(14) Neil A, et al. Reductions in all-cause, and coronary mortality in statin-treated patients with heterozygous familial hypercholesterolaemia. *European Heart Journal* 2008; 29(21): 2625-33
(15) Marks D et al.. Cost effectiveness analysis of different approaches of screening for familial hypercholesterolaemia. *British Medical Journal* 2002; 324(7349): 1303

**Reference**	MR/T017384/1
**Applicants**	Professor Nadeem Qureshi and Professor Anis Safura Ramli
**Title**	Reducing Premature Coronary Artery Disease in Malaysia by early identification of Familial Hypercholesterolaemia
**Organisations**	University of Nottingham and MARA University of Malaysia (UiTM) and National University of Malasyia (UKM)
**Median Score**	8
**Decision**	Award
**PM**	Melissa Lennartz-Walker

FEEDBACK

Dear Professor Nadeem Qureshi and Professor Anis Safura Ramli,

Thank you for submitting the above proposal for consideration the UK-Malaysia Joint Partnership on Non-Communicable Diseases panel.

The panel's comments and questions in relation to your proposal are below.

The panel noted that:

- The topic of the research was relevant and timely, with familial hypercholesterolemia (FH) is more common in Malaysians compared to European-origin populations.
- The applicants made a very strong case for the need to develop a robust system of detecting FH in the primary care setting in Malaysia, with potential impact. FH is one of the well-recognised causes of premature coronary artery disease, and early detection of FH may have a significant impact on affected patients.
- The approach, notably to validate the FAMCAT algorithm for detection of (FH) compared to S-B and DLCC criteria, and also to utilise the web-based tools in Malaysian primary care clinics and compare with genetic testing was noted as innovative.
- The suggested team was strong with relevant expertise and a good track record.
- Good value for money was identified here.

However, the Panel also noted that:

- The study design was not clear and would have benefitted from more structure and justification.
- Details of the genetic analysis were lacking (i.e. variant annotation/confirmation of the pathogenicity of the new variants). This is particularly relevant as a significant proportion of variants may represent new mutations (not previously observed in the population of white European ethnicity). Additionally, the sequencing strategy was focused on the candidate genes. A more agnostic approach may have been more beneficial (i.e. resulting in uncovering new genes for FH in this population). However, these concerns were well addressed in the rebuttal.

Yours Sincerely,

Melissa Lennartz-Walker
Newton Fund Programme Manager

Email: melissa.lennartz-walker@mrc.ukri.org

Head of Department
Research and Innovation (Admin)
University of Nottingham
King's Meadow Campus
Nottingham United Kingdom
NG7 2NR

Grant Ref: MR/T017384/1

Date: 26 November 2019

Dear Head of Department

GRANT OFFER: Research Grant, Research Grants
GRANT TITLE: Reducing Premature Coronary Artery Disease in Malaysia by early identification of Familial Hypercholesterolaemia

The MRC is offering a grant towards the cost of the above project, subject to the terms and conditions set out below.

Return of the 'Offer Acceptance' will be taken as acceptance of the grant on the terms stated. If you are unable to accept the grant you should return a 'Decline' confirmation as soon as possible. Upon receipt of the 'Offer Acceptance' a 'Start Confirmation' request will be issued.

Grants are cash limited and expenditure against the grant must not exceed the value awarded apart for reasons stated in the standard terms and conditions.

Please note copies of this letter have not been sent to the grant holder and co-investigators (as appropriate); it is your responsibility to distribute copies as is necessary.

Yours faithfully

Grants Pre Award Team
RCUK Grants
A service provided on behalf of MRC

Organisation: University of Nottingham

Grant Holder: Professor Nadeem Qureshi

Grant Title: Reducing Premature Coronary Artery Disease in Malaysia by early identification of Familial Hypercholesterolaemia
Starts: 31 December 2019 **Ends:** 30 December 2021 **Duration:** 24

GRANT VALUE

Funds Awarded

	Authorised FEC (£)			RC Contribution (£)			
	net	Indexation	Total	net	Indexation	Total	% FEC
DI - Staff	59,321	723	60,044	47,457	578	48,035	80
DI - T&S	27,200	331	27,531	21,760	265	22,025	80
DI - Other Costs	20,000	244	20,244	16,000	195	16,195	80
DA - Investigators	34,263	417	34,680	27,410	334	27,744	80
DA - Estate Costs	20,751	253	21,004	16,601	202	16,803	80
Indirect - Indirect Costs	84,454	1,029	85,483	67,563	823	68,386	80
**Total Value of Award**	**245,989**	**2,997**	**248,985**	**196,791**	**2,397**	**199,188**	

Cost of Access to Facilities 0
(Funds not awarded to Grant Holding Organisation)

STAFF

Staff Summary

	Authorised FEC net	RC Contribution net	Number Of Staff Months
Investigator	34,263	27,410	4
Researcher	59,321	47,457	17

Staff and DI Investigator Details

Start Date	End Date	Duration	FTE Percent	Name or Post Identifier	Summary Fund Heading	Authorised Cost (Excluding Indexation)
31 December 2019	30 December 2021	24	50	Quantitative Research Fellow	Directly Incurred	40099.00
31 December 2019	30 December 2021	24	20	Qualitative Research Fellow	Directly Incurred	19222.00

DA Investigator Details

Average Hours/week	Name or Post Identifier
0	Dr H Abdul Hamid
0	Dr S Abdul Razak
0	Dr A Al-Khateeb
0	Dr S Badlishah Sham
0	Dr N Baharudin
1.1	Professor J Kai

Average Hours/week	Name or Post Identifier
1.1	Professor J Leonardi-Bee
0	Dr M Mohamed-Yassin
0	Dr M Mohamed-Yassin
0	Professor H Mohd Nawawi
0	Dr N Nasir
1.9	Professor N Qureshi
0	Professor A Ramli
1.9	Dr S Weng

EQUIPMENT DETAILS

Description	Delivery Date	Country Of Origin	Total Value

FACILITY AND SERVICE DETAILS

Facility	Cost of Access	Number of Units

PROJECT PARTNERS

Organisation	Department	Last Name	First Name	In Kind Value (£)	Monetary Value (£)

GRANT ADDITIONAL INFORMATION

GRANT CONDITIONS

GRANT ADDITIONAL CONDITIONS: NEWTON FUND

This project has been recommended for funding under the Newton Fund UK-Malaysia: Joint Partnership call on Non-Communicable Diseases. This project is funded by the Newton Fund Programme, and this grant is awarded by the Medical Research Council on behalf of UK Research and Innovation.

GAC NF 1: ODA compliance
The Newton Fund is part of the UK's Official Development Assistance (ODA). Its aim is to develop science and innovation partnerships that promote the economic development and welfare of developing countries. The investigators must ensure the research that is undertaken as part of this grant is compliant with ODA rules and regulations as set out by the OECD. In the event that the research is deemed to no longer comply with ODA rules and regulations the Medical Research Council reserve the right to terminate the grant.

Further information on ODA guidance can be found at:
https://www.ukri.org/files/legacy/international/gcrfodaguidance-pdf/

In addition to the requirements set out in RGC 5, any proposed changes to the award that could affect the ODA compliance of the research must be reported to the Medical Research Council at the earliest opportunity via the Grant Maintenance facility in Je-S. The Medical Research Council will then consider whether such changes are permissible and inform the Research Organisation of their decision.

Changes that should be reported include but are not limited to any proposed changes to the Principal Investigator, Co-Investigator, project partners, field study sites, country of focus and objectives.

GAC NF 2: Acknowledgements and reporting
In addition to the provisions in RGC22, the investigators must acknowledge the Newton Fund and the Medical Research Council / UK Research and Innovation in any publications, web pages or events associated with this grant.

Investigators must assist the Medical Research Council / UK Research and Innovation with any additional reporting requirements requested by the Department for Business, Energy and Industrial Strategy.

GAC NF 3: Starting Procedures

This grant has a FIXED start date of 31st December 2019. The start of the grant may NOT be delayed beyond or precede this date.

Please note that due to the fixed start date, the normal three month start period rules outlined in UKRI Terms and Conditions RGC5 do not apply to this project.

GAC NF 4: Ethical requirements
Research must meet the RCUK Research Governance guidelines outlined in RGC2. For clinical studies involving human participants and/or patients, appropriate consent must be obtained. Additionally, any research undertaken outside the UK must have both UK and respective country ethical approvals.

When collaborating with other laboratories, or where animal facilities are provided by third parties, researchers and the local ethics committee in the UK should satisfy themselves that welfare standards consistent with the principles of UK legislation (e.g. the ASPA) and set out in this guidance are applied and maintained.

https://mrc.ukri.org/funding/guidance-for-applicants/5-ethics-and-approvals/

The Principal Investigator/ Research Organisation must be prepared to furnish the Medical Research Council / UK Research and Innovation with a copy of the ethical approval, and any correspondence with the committees, if requested by the Council. The principal investigator must notify the Medical Research Council / UK Research and Innovation if a regulator or a research ethics committee requires amendments that substantially affect the research question, methodology or costs to the extent that the project is no longer the same as that approved for funding by the Medical Research Council / UK Research and Innovation.

GAC NF 5: Government support
This award is dependent on continuing Government commitment for this initiative. In the event that this support is withdrawn, the Medical Research Council reserve the right to terminate the award.

GAC NF 6: Requests for extensions to awards
Due to financial restraints of the Newton Fund Programme, grant extensions will only be considered under exceptional circumstances (in line with the Equality Act 2010) and will require the Medical Research Council agreement on a case-by-case basis. The Research Organisation remains responsible for compliance with the terms of the Equality Act 2010 including any subsequent amendments introduced while work is in progress; and for ensuring that the expectations set out in the RCUK statement of expectations for equality and diversity are met.

GAC NF 7: Transfer of funds to UK and overseas organisations
In accordance with RGC1, it is important to highlight that the Research Organisation awarded the grant is responsible for the conduct and administration of the grant during the life time of the award (from award, during the grant and on completion). It is accountable for the effective use of public funds, and must therefore ensure that all grant monies are subject to proper financial management processes. It is the Research Organisation's responsibility to ensure that, where funds are transferred to other organisations in the UK and abroad, expenditure is subject to robust controls to ensure value for money and propriety and that all costs should be fully vouched and maintained for possible inspection and checks by, or on behalf of, the funding organisation.

This award has therefore been made on the basis that if any funds are transferred to another UK or overseas organisation then the Research Organisation must undertake due diligence checks to ensure that the funding will be appropriately used (as set out above). The Research Organisation may be asked to provide evidence that where funds have been transferred they have undertaken appropriate due diligence to ensure that any risks are recognised, understood and treated as necessary. The Research Organisation may be asked to provide additional information on how the due diligence checks were carried out.

Please refer to the Medical Research Council for any specific guidance.

GAC NF 8: Collaboration agreement
In accordance with RGC 20, a Collaborative Agreement is required for this project. This must be in place within six months of the start of the project.

As the grant is associated with more than one research organisation the basis of collaboration between the organisations, including the allocation of resources throughout the project and ownership of intellectual property and rights to exploitation, is required to be set out in the formal collaboration agreement. It is the responsibility of the lead Research Organisation to put such an agreement in place within six months of the start of the project. The terms of collaboration agreements must not conflict with the Research Councils' terms and conditions.

GAC NF 9: Change in Principal Investigator
This award has been made on the basis of the named individual's suitability to undertake the research and that they have the expertise and experience to lead the project to a successful conclusion, in accordance with its research objectives. This award is made on condition that any request to change the principal investigator/ Co-Investigator will require the Medical Research Councils prior approval. Requests for a change in principal investigator are to be submitted via the Grant Maintenance facility in Je-S. The Medical Research Council will then consider and inform the Research Organisation of their decision.

CALL CONDITIONS

RESEARCH COUNCIL CONDITIONS

SCHEME CONDITIONS

UK RESEARCH AND INNOVATION fEC GRANTS

STANDARD TERMS AND CONDITIONS OF GRANT

UK Research and Innovation fEC Grants Standard Terms and Conditions of Grant
The Standard Terms and Conditions of Grant apply to Research Grants and Fellowships, costed and funded on a Full Economic Costs basis (fEC) and calculated according to the Transparent Approach to Costing (TRAC) or an equivalent methodology, awarded by the following seven UK Research and Innovation (UKRI) Councils:

Arts and Humanities Research Council (AHRC)
Biotechnology and Biological Sciences Research Council (BBSRC)
Economic and Social Research Council (ESRC)
Engineering and Physical Sciences Research Council (EPSRC)
Medical Research Council (MRC)
Natural Environment Research Council (NERC)
Science and Technology Facilities Council (STFC)

Application of Standard Terms and Conditions of Grant
In these Standard Terms and Conditions of Grant, the words "We", "Our" or "Us" refer to the relevant Council of UKRI awarding the Grant and "You" or "Your" refer to the Research Organisation in receipt of the Grant. Other key terms used in these Standard Terms and Conditions of Grant are set out in the Definitions attached at Annex A.

These Standard Terms and Conditions of Grant, together with any applicable Specific Terms and Conditions of Grant required by an individual Council of UKRI comprise the Grant Terms and Conditions on which UKRI awards the Grant to the Research Organisation. Specific Terms and Conditions of Grant will be set out in the Grant Offer Letter.

These Grant Terms and Conditions should be read in conjunction with the sources outlined in Annex B, in the event of any conflict the terms of these Conditions should prevail.

Use of Grant Proposal Information
UK Research and Innovation (UKRI) handles all personal data in accordance with current UK data protection legislation and the EU General Data Protection Regulation (GDPR) where appropriate.
It is the responsibility of the Research Organisation to ensure that both students it funds from UKRI funding and individuals who receive grant funding, or who are later involved in the award, are made aware of how personal data may be used by both UKRI and the Research Organisation. This includes information relating to groups such as students, supervisors, project partners, investigators, named researchers and support staff.

To meet UKRI's obligations for public accountability and the dissemination of information, contents of funded research proposals will also be made available on the Councils' websites and other publicly available sources. As a condition of funding, UKRI may use the data to publish information on awards made. We may also share information with third parties to support, for example, open access publication and reporting outcomes via Researchfish. This includes data submitted through Je-S Student Details (SD).
UKRI is also subject to the UK Freedom of Information Act (2000) and the Environmental Information Regulations (2004) and may be required to release grant information on request, subject to appropriate exemptions.
Further information is provided by the UKRI Use of grant proposal information addendum (www.ukri.org/files/funding/tcs/grants-addendum-pdf/) and via the UKRI Privacy Notice (www.ukri.org/privacy-notice/).

Standard Terms and Conditions of Grant

RGC 1 Variation to Terms and Conditions
UKRI reserves the right to amend and vary these Standard Terms and Conditions of Grant and any Specific Terms and Conditions of Grant or applicable policies at any time. The latest version of the Standard Terms and Conditions of Training Grant are available on the UKRI website at: https://www.ukri.org/funding/information-for-award-holders/grant-terms-and-conditions/

RGC 2 Accountability & Responsibilities of the Research Organisation
RGC 2.1 You are responsible for ensuring that the Project carried out by You, the Grant Holder and any Research Workers or other Third Parties, comply with these Standard Terms and Conditions of Grant and any Specific Terms and Conditions of Grant.

RGC 2.2 You must ensure that the Project is carried out in accordance with all applicable ethical, legal and regulatory requirements including but not limited to relevant provisions of the General Data Protection Regulation, the Data Protection Act 2018, the Bribery Act 2010, the Fraud Act 2006, the Equality Act 2010 and the Modern Slavery Act 2015.

RGC 2.3 You must ensure that Your use of the Grant complies with European Union State Aid law and You acknowledge that if

You breach State Aid law, UKRI may be required to recover some or all Grant funding, together with interest.

RGC 2.4 You are accountable for the conduct of the Project including the conduct of the research, the use of public funds and the proper financial management of the Grant in accordance with these Standard Terms and Conditions of Grant and any Specific Terms and Conditions of Grant, whether the Project is carried out by You or the Grant Holder, Research Workers or other Third Party.

RGC 2.5 You must ensure that the Grant is spent in a way that is consistent with the purpose and conditions set out in the Offer Letter.

RGC 2.6 You must carry out appropriate due diligence on any Third Parties used to deliver any part of the Project and shall ensure in particular, that such Third Parties comply with these Standard Terms and Conditions of Grant and any Specific Terms and Conditions of Grant. At UKRI's request, You must provide details of expenditure of the Grant by any Third Party. Where all, or part, of the Project is carried out by Third Parties based overseas, You must follow the UKRI International Due Diligence Guidance at: http://www.ukri.org/files/funding/due-diligence-guidance-for-ukros-pdf/

RGC 2.7 You must ensure that any part of the Full Economic Cost not funded by the Grant is committed to the Project before it starts.

RGC 2.8 You must have adequate business continuity plans in place to ensure minimum operational interruptions to the Project.

RGC 2.9 In order to foster a research culture which values, recognises and supports public engagement, You must adopt the principles, standards and good practice for public engagement with research set out in the 2010 Concordat for Engaging the Public with Research at: https://www.ukri.org/public-engagement/research-council-partners-and-public-engagement-with-research/embedding-public-engagement/

RGC 2.10 You must notify UKRI of any changes to Your constitution, legal for, membership structure (if applicable) or ownership, including those that might affect Your eligibility to hold the Grant, or to deliver the Project or any other changes which affect Your ability to comply with the Grant Terms and Conditions.

RGC 2.11 You must ensure that the requirements of the Employing Organisation under the UK Policy Framework for Health and Social Care Research (or equivalent) are met for research involving National Health Service (or equivalent) patients, their organs, tissues or data, and that the necessary arrangements are in place with partner organisations. Where You also accept the responsibilities of a Sponsor (as defined in the Policy Framework), You must also ensure that the requirements for Sponsors are met.

RGC 2.12 Peer review is an integral part of the application process and ensures research of the highest calibre is funded. Investigators and named Researchers on this Grant are expected to make all reasonable efforts to undertake the peer review of proposals for UKRI when invited to do so, unless there is a conflict of interest or the proposal is outside of their area of expertise.

RGC 2.13 By accepting this Grant You are confirming that the Grant Holder has not already received competitively obtained research or support funding from any source, for the same research Project that this Grant has been awarded by Us to support. We reserve the right to terminate the Grant should We find that the Grant Holder has been or is in receipt of the aforementioned duplicate funding, either before or during the Grant Period.

RGC 3 Research Governance
RGC 3.1 Research Ethics, Misconduct and Conflicts of Interest
RGC 3.1.1 You are responsible for ensuring that ethical issues relating to the Project are identified and brought to the attention of the relevant approval or regulatory body. Before any such work requiring approval begins, approval must have been granted by the relevant body.

RGC 3.1.2 You must follow Our Policy and Guidelines on Governance of Good Research Conduct at: https://www.ukri.org/about-us/policies-and-standards/research-integrity/ and ensure that the requirements set out in the Concordat to Support Research Integrity (2012) are met. In particular, You are responsible for ensuring all necessary permissions are obtained before the Project begins, that there is clarity in roles and responsibility among Grant Holders, Research Workers, and Third Parties, as well as investigating and reporting unacceptable research conduct. Any potential conflicts of interest in research identified at the point of application must be declared to Us and subsequently managed.

RGC 3.2 Use of Animals in Research
You must comply with the provisions of the Animals (Scientific Procedures) Act 1986, and any amendments, where applicable and ensure that all necessary licences are in place before any work requiring approval takes place. You should also follow the guidance set out in "Responsibility in the use of animals in bioscience research" at: https://www.nc3rs.org.uk/responsibility-use-animals-bioscience-research

RGC 3.3 Health and Safety
You are responsible for ensuring a safe working environment for all individuals associated with the Project, both on and off-site, and for meeting all regulatory and legislative health and safety requirements.

We reserve the right to require You to undertake a safety risk assessment in individual cases where health and safety may be an issue, and to monitor and audit the actual arrangements made. In the event of a serious incident (e.g. death) we require that you inform us for risk purposes.

RGC 3.4 Equality, Diversity and Inclusion
You are expected to ensure that equality, diversity and inclusion is considered and supported at all stages throughout the

performance of the Project, in alignment with Our policies and principles at: https://www.ukri.org/about-us/policies-and-standards/equality-diversity-and-inclusion/ for equality, diversity and inclusion. Your approach to supporting equality, diversity and inclusion is expected to exceed all relevant legal obligations, including but not limited those of the Equality Act 2010.

RGC 3.5 Safeguarding
All relevant safeguarding legislation must be adhered to, We particularly draw your attention to child protection legislation and the Modern Slavery Act 2015. You must have sufficient policies and/or processes in place in order to foster Safeguarding.

RGC 3.6 Bullying and Harassment
You must have clear, well-publicised policies, processes and training in place consistent with good practice as recommended by the Advisory, Conciliation and Arbitration Service's (ACAS) 'Bullying and Harassment in the Workplace: A Guide for Managers and Employers'.

RGC 4 Use of Grant
RGC 4.1 We reserve the right to vary the value of the Grant during its lifetime in accordance with the GDP Deflators published by HM Government or to take into account any other Government decisions affecting the funding available to UKRI.

RGC 4.2 With the exception of RGC 4.3, Directly Incurred and Exceptions funds must not be used to meet the costs of an activity that will fall outside the Grant Period.

RGC 4.3 Expenditure may be incurred prior to the start of the Grant and be subsequently charged to the Grant, provided that it does not precede the date of the Offer Letter.

RGC 4.4 Transfers of funds between fund headings are permitted only within and between Directly Incurred and Exceptions costs, excluding equipment, at the rate applicable for the heading as set out in the award letter. Funds may only be transferred into studentship stipend or fees to supplement an existing studentship post on the Grant. You must not transfer funds to create new posts without prior approval from UKRI. Directly Incurred and Exceptions funds must not be used to meet costs on any other Grant or activity.

Funds can only be transferred and used to meet the cost of activity or activities that meet the agreed aims and objectives of the project. While approval does not need to be sought from Us for transfer of funds (excluding the creation of new posts), We reserve the right to query any expenditure outlined in the Final Expenditure Statement which has not been incurred in line with the Standard Terms and Conditions of Grant and any Specific Terms and Conditions of Grant.

RGC 4.5 Costs associated to Students must not be charged to the Grant. These costs must be met by other resources held by You, which can include UKRI Training Grants if the student holds a UKRI studentship. Students are able to undertake paid work within the institution as casual assistance, this should be evidenced with a clear audit trail and should not form part of the formal studentship training.

RGC 5 Starting Procedures
RGC 5.1 You must formally accept the Grant by completing and returning the Offer Acceptance within 10 working days of the issue of the Offer Letter.

RGC 5.2 You must submit the Start Confirmation within 42 (calendar) days of the Project starting. The date entered on the Start Confirmation will be the Official Start Date of the Grant. The Official Start Date may be delayed by up to 3 months from the start date shown in the Offer Letter, but the duration of the Grant will remain unchanged. The Grant may lapse if the Project is not started within 3 months of the start date in the Offer Letter. The start of the Grant may precede the start date shown in the Offer Letter, but must not be earlier than the issue date of the Offer Letter itself.

RGC 6 Extensions
RGC 6.1 The duration of the Grant ("Grant Period") may be extended after the Official Start Date by up to 12 months without additional funding subject to Our prior written approval. For further information, see the UKRI fEC Grant Guidance document.

RGC 6.2 For Fellowship Grants, the Grant Period may also be extended to cover familial leave, extended jury service or paid sick leave after the Official Start Date for a period in line with the Terms and Conditions of the Fellow's employment. For further information, see the UKRI fEC Grant Guidance document.

RGC 7 Monitoring
RGC 7.1 Changes to Project
You must inform and consult Us if there are any significant changes that may affect the progress or delivery of the Project. No substantive changes to the experimental design of a project involving the use of animals or human participation, which might affect the ethical characteristics of the award, are permitted without the prior approval of UKRI.

If You propose to make significant changes to the Project, UKRI may require revised proposals for its approval and reserves the right to make a new Grant in place of the existing Grant, or to revise, retain or terminate the existing Grant.

RGC 7.2 Transfer of a Grant to another Research Organisation
RGC 7.2.1 The Grant may be transferred to another eligible organisation, providing that it can provide a suitable environment to enable the project to be successfully completed; this will be subject to prior written approval of UKRI. Written agreement to this is required from both the relinquishing and receiving organisations.

RGC 7.2.2 Grant funding will not be revised following transfer. The receiving organisation must confirm that it will provide any additional resources needed to complete the project by returning an Offer Acceptance.

RGC 7.3 Change of Grant Holder
RGC 7.3.1 For Research Grants, You must submit any proposed changes of Grant Holder to UKRI for approval via the Grant Maintenance facility in Je-S.

RGC 7.3.2 For Fellowship Grants, changes to the Grant Holder are not permitted. In the event of the research fellow's resignation or other termination of their employment, the Grant will terminate automatically.

RGC 7.4 Research Monitoring and Evaluation
RGC 7.4.1 You must use Our nominated online system to submit information for monitoring and evaluation purposes on the outputs and outcomes and impacts of the Project during and for some years after the expiry of the Grant Period. Further information on reporting requirements can be found on the UKRI website: https://www.ukri.org/funding/information-for-award-holders/research-outcomes/help-and-guidance/. Failure to comply with the reporting requirements will result in suspension of Grant payments and no further proposals will be considered by UKRI where the Grant Holder is named as the Principal or Co-Investigator.

RGC 7.4.2 Exceptionally We may require a separate End of Award Report on the conduct and outcome of the Project. If required You must submit the report within 3 months of the end of the Grant Period. No further application from a Grant Holder will be considered while an End of Award Report is overdue.

RGC 7.4.3 We reserve the right to call for periodic updates on the Project's progress or to visit the Project team, or request participation in evaluation studies. The Grant Holder must make all reasonable efforts, if so invited, to respond to requests for information or to attend events or activities organised by UKRI concerning the research undertaken, including requests or events after the end of the Grant Period.

RGC 7.5 Disclosure and Inspection
RGC 7.5.1 We shall be entitled to inspect any financial or other records and procedures associated with the Grant as are reasonably required to verify the regularity and propriety of Grant expenditure, or to appoint any other body or individual for the purpose of such inspection. This includes expenditure by Third Parties.

RGC 7.5.2 If We request it, You must provide a statement of account for the Grant, independently examined by an auditor who is a member of a recognised professional body, certifying that the expenditure has been incurred in accordance with the Grant Terms and Conditions.

RGC 7.5.3 You must report to us any investigations and their outcomes into research misconduct associated with the Grant in advance of any enquiry whether informal or formal, and upon request, provide information on Your management of research integrity and ethics as described at: www.ukri.org/about-us/policies-and-standards/research-integrity/. In addition, You must provide details of any retractions or withdrawal of submissions/publications, any allegations, proven or not, of cases of fraud and any other complaint or investigation into dishonesty, fraudulent activities or business misconduct, by any regulatory body or the police into Your activities or those of Your staff.

RGC 7.5.4 We will undertake periodic reviews of Research Organisations within the Funding Assurance Programme to seek assurance that Grants are managed in accordance with the Terms and Conditions under which they are awarded.

RGC 8 Staff
RGC 8.1 Employment
You are wholly responsible for staff funded from the Grant, including Research Fellows, and accept all duties owed to and responsibilities for these staff, including, without limitation, their terms and conditions of employment, and their training and supervision, arising from the employer/employee relationship. You must appoint a Research Fellow as an employee for the full duration of the award.

RGC 8.2 Career Development
You are expected to adopt the principles, standards and good practice for the management of research staff set out in the 2008 Concordat to Support the Career Development of Researchers, including any subsequent amendments.

RGC 8.3 Maternity, Paternity, Adoption and Parental Leave
RGC 8.3.1 At the end of the Grant Period We will reimburse costs incurred by You to cover any additional net parental leave costs that cannot be met within the announced grant cash limit including Statutory Maternity, Paternity and Adoption Pay for staff, within the Directly Incurred and Exceptions fund headings (excluding Principal and Co-investigators, unless they are Research Fellows or Researcher Co-Investigators). This will be payable only for the percentage of time that the staff are contracted on the Grant.

RGC 8.3.2 Within the announced grant cash limit, the Grant may be used to meet the costs of making a substitute appointment and/or extending the Grant to cover a period of parental leave for staff within the Directly Incurred and Exceptions fund headings (as outlined above). Directly Allocated and Indirect funds will not be increased as a result of such extensions.

RGC 8.3.3 You will be responsible for any liability for parental leave pay for staff supported by the Grant outside the original Grant Period.

RGC 8.3.4 Fellows are entitled to take parental leave in accordance with the terms and conditions of their employment. We will consider requests for a Fellowship Grant to be placed in abeyance during the absence of the Research Fellow for parental leave, and the period of the Fellowship extended by the period of leave. We will also consider requests to continue the Fellowship on a flexible or part-time basis to allow the Research Fellow to meet caring responsibilities.

RGC 8.4 Sick Leave
RGC 8.4.1 At the end of the Grant Period, We will reimburse You for any additional net sick leave costs that cannot be met within the announced Grant cash limit for staff within the Directly Incurred and Exceptions fund headings (excluding Principal and Co-

Investigators, unless they are Research Fellows or Researcher Co-investigators), except where You have already recovered these costs by claiming Statutory Sick Pay from HMRC. This will be payable only for the percentage of time that the staff are contracted on the Grant.

RGC 8.4.2 Within the announced grant cash limit, the Grant may be used to meet the costs of making a substitute appointment and/or extending the Grant to cover a period of sick leave for staff within the Directly Incurred and Exceptions fund headings (as outlined RGC 8.4.1). Directly Allocated and Indirect funds will not be increased as a result of such extensions.

RGC 8.4.3 You will be responsible for any liability for sick leave pay for staff supported by the Grant outside the original Grant Period.

RGC 8.4.4 Where there is a continuous period of sick leave in excess of 3 months, You may request approval for a substitute appointment to safeguard progress on the Project. Where a Research Assistant has been on sick leave in excess of 3 months, You must comply with all obligations to consider reasonable adjustments before making a substitute appointment. Where a Research Assistant has been on sick leave for an aggregate (not necessarily continuous) period in excess of 3 months, where this is due to a single condition or a series of related conditions, You may request an extension to the duration of the project.

RGC 8.4.5 Fellowship Grants: Fellows are entitled to take sick leave in accordance with the Research Organisation's terms and conditions. If requested, consideration will be given to allowing a fellowship grant to be placed in abeyance during the absence of the Research Fellow due to sick leave, and the period of the fellowship extended by the period of sick leave. The additional salary costs for the fellow (pro rata to their percentage FTE on the fellowship) should be claimed, as necessary, at the end of the extended period

RGC 9 Equipment
RGC 9.1 Procurement of Equipment
The procurement of equipment, consumables and services, including maintenance, must comply with all relevant national and EU legislation. For contracts over £25,000, excluding VAT, professionally qualified procurement staff must be consulted before the procurement process begins. Any proposal to purchase equipment in the last 6 months of the Grant must be pre-approved by UKRI.

RGC 9.2 Ownership of Equipment
You must inform us if the need for the equipment diminishes substantially or it is not used for the purpose for which it was funded during the Grant Period. We reserve the right to determine the disposal of such equipment and to claim the proceeds of any sale. Any proposal to transfer ownership of the equipment during the period of the Grant requires the prior approval by UKRI.

RGC 9.3 Equipment Data
All new equipment purchased over £138,000 (£115,000 ex VAT) must be registered on the "Equipment.data" national database.

RGC 10 Financial Reporting
RGC 10.1 You are accountable for funds dispersed and are responsible for the timely and accurate submission of all expenditure reports required under the Terms and Conditions of Grant, including the submission of an expenditure statement within 3 months of the end of the Grant Period. We are entitled to require You to provide supplementary information in support of an interim or final expenditure statement. Once an expenditure statement has been received and the expenditure incurred has been reconciled against payments made, it will be considered as final. Any unspent funds will be recovered.

RGC 10.2 You must retain all accounting information relating to the Grant for the current financial year plus the subsequent six years after the submission date of the final expenditure statement.

RGC 10.3 If We send an Annual Statement to return showing payments made by UKRI during the previous financial year for all the Grants You hold, You must complete and return the statement by the specified deadline.

RGC 11 Sanctions
RGC 11.1 We reserve the right to impose financial sanctions and/or additional measures if You do not comply with Your obligations as set out in these Standard Terms and Conditions of Grant and any Specific Terms and Conditions of Grant.

RGC 11.2 If the End of Award Report (if required) or the Financial Expenditure Statement is not received within 3 months of the end of the Grant Period, UKRI will recover 20% of expenditure incurred on the Grant. All payments will be recovered if the report or statement is not received within 6 months of the end of the Grant. You may appeal against a sanction, but must do so within 60 days of the pay run in which the sanction was imposed.

RGC 11.3 Where an Organisation is deemed to be non-compliant in the application of TRAC, a minimum sanction of 75% of the non-compliant rate may be applied, where an Organisation is applying rates which are materially inaccurate (>10% variance on any single rate). These sanctions would only apply to future applications, until a time that UKRI Funding Assurance are satisfied that remedial measures are implemented.

RGC 12 Exploitation and Impact
RGC 12.1 Unless otherwise agreed, all intellectual property shall belong to the party that generates them. Where the Grant is associated with more than one Research Organisation and/or other project partners, the basis of collaboration between the organisations including ownership of intellectual property and rights to exploitation, is expected to be set out in a formal collaboration agreement.

RGC 12.2 You are responsible for ensuring that all parties engaged in the research make every reasonable effort to ensure that the intellectual assets obtained in the course of the research, whether protected by intellectual property rights or not, are used to the benefit of society and the economy.

RGC 12.3 In individual cases, We reserve the right to retain ownership of intellectual assets, including intellectual property (or assign it to a third party under an exploitation agreement) and to arrange for it to be exploited for the national benefit and that of the Research Organisation involved.

RGC 12.4 The Grant Holder shall, subject to the procedures laid down by the Research Organisation, publish the results of the research funded by the Grant in accordance with normal academic practice and Our policy on Open Access: https://www.ukri.org/files/legacy/documents/rcukopenaccesspolicy-pdf/. Other forms of media communication, including media appearances, press releases and conferences, must acknowledge the support received from Us, quoting the Grant reference number if appropriate.

RGC 13 Disclaimer
RGC 13.1 UK Research and Innovation accepts no liability, financial or otherwise, for expenditure or liability arising from the research funded by the Grant except as set out in these Terms and Conditions, or otherwise agreed in writing.

RGC 13.2 UKRI reserves the right to amend the payment profile at its discretion. You will be advised, in advance, of any such change. Changes to payment profiles may affect the overall value of the Grant.

RGC 13.3 UKRI reserves the right to terminate the Grant at any time, subject to reasonable notice and to any payment that We agree may be necessary to cover outstanding and unavoidable commitments. If a Grant is terminated or reduced in value, no liability for payment, redundancy or any other compensatory payment for the dismissal of staff funded by the Grant will be accepted, but, subject to the provisions of RGC 10 Financial Reporting, negotiations will be held with regard to other contractual commitments and concerning the disposal of assets acquired under the research grant.

RGC 13.4 Where studies are carried out in an NHS Trust or equivalent, the Trust or equivalent has a duty of care to its patients. UK Research and Innovation does not accept liability for any failure in the Trust's duty of care, or any negligence on the part of its employees.

RGC 14 Status
RGC 14.1 The Terms and Conditions of Grant which include these Standard Terms and Conditions of Grant and the Specific Terms and Conditions of Grant will be governed by the laws of England and Wales and all matters relating to the Terms and Conditions will be subject to the exclusive jurisdiction of the courts of England and Wales.

RGC 14.2 If any provision of these Terms and Conditions is found by a court or other legitimate body to be illegal, invalid or unreasonable, it will not affect the remaining Terms and Conditions which will continue in force.

RGC 14.3 The Terms and Conditions of Grant contain the whole agreement between UKRI and the Research Organisation in relation to the Grant and neither party intends that any of these Terms and Conditions should be enforceable by any third party.

Annex A
Definitions

Co-Investigator: A person who assists the Grant Holder in the management and leadership of the Project.

Council: Any of the bodies listed under the Introduction.

Directly Allocated Costs: Costs of resources used by the Project that are shared by other activities. They are charged on the basis of estimates rather than actual costs and do not represent actual costs on a project by project basis.

Directly Incurred Costs: Costs that are explicitly identifiable as arising from the conduct of the Project which are charged as the cash value actually spent and are supported by an audit record.

End of Award Report: A report which the Grant Holder must provide at the end of the Grant Period, detailing the outputs, outcomes and impacts of the project to date.

Exceptions: Directly Incurred Costs that Councils fund at 100% of fEC subject to actual expenditure incurred, or items that are outside fEC.

Fellowship Grant: An award made through a fellowship competition providing a contribution to the support of a named individual. It covers the cost of the time dedicated by the fellow to their personal research programme, and may or may not include research support costs.

Full Economic Costs (fEC): A cost which, if recovered across an organisation's full programme, would recover the total cost (direct, indirect and total overhead) including an adequate recurring investment in the organisation's infrastructure.

Funding Assurance Programme: A programme of visits and office based tests by UKRI to seek assurance that grant funds are used for the purpose for which they are given and that grants are managed in accordance with the terms and conditions under which they are awarded Gran Support for a proportion of the full economic costs of the Project. A Grant may be either a Research Grant or a Fellowship.

Grant Holder: The person to whom the Grant is assigned and who has responsibility for the intellectual leadership of the Project and for the overall management of the research funded by the Grant. The Grant Holder is either the Principal Investigator (in the case of a Research Grant) or a Research Fellow (in the case of a Fellowship Grant).

Grant Period: The duration of time between the Project start and end date.

Grant Terms and Conditions: The Standard Terms and Conditions of Grant together with the Specific Terms and Conditions of Grant that together comprise the basis on which the Grant is awarded to the Research Organisation.

Indirect Costs: Non-specific costs charged across all projects based on estimates that are not otherwise included as Directly Allocated Costs. They include the costs of the Research Organisation's administration such as personnel, finance, library and some departmental services.

Je-S: Joint Electronic Submissions system used for the submission of Grant related information.

Offer Acceptance: A document to be completed and returned by the Research Organisation either accepting or declining the Grant.

Grant Offer Letter / Offer Letter: An official document setting out specific details of the Grant, including the Project start and end date, Grant value and any Specific Terms and Conditions of the Grant as required by the relevant Council.

Official Start Date: The official start date of the Grant, as set out in the Start Confirmation.

Project: The project funded by the Grant as set out in the Offer Letter.

Research Grant: A contribution to the costs of the research Project which has been assessed as eligible for funding through the procedures established by the relevant Council.

Research Organisation (RO)/Grant Awardee: The organisation to which the Grant is awarded and which takes responsibility for the management of the Project and accountability for funds provided.

Research Worker: Any person or third party working in any capacity on the Project.

Specific Terms and Conditions of Grant/Specific Conditions: The specific conditions of grant required in addition to the Standard Terms and Conditions on a Grant by an individual Council of UKRI.

Standard Conditions of Grant/Standard Conditions: The Standard Terms and Conditions of Grant published on UKRI's website at: www.ukri.org/funding/information-for-award-holders/grant-terms-and-conditions/

Start Confirmation: Confirmation of the date on which the Project commences, as notified by the Research Organisation to UKRI.

Studentship: The term used for the funding award made by a Research Organisation to a student for the purpose of undertaking postgraduate training leading to the award of a postgraduate degree.

Third Party: Any person/organisation to which the award holding RO passes on any of the Grant funds awarded by the Council.

Transparent Approach to Costing (TRAC): An agreed methodology used by universities and other higher education bodies for calculating full economic costs.

Annex B

Information Sources

These Grant Terms and Conditions should be read in conjunction with the following sources. In the event of any conflict the terms of these Conditions should prevail:

1) UKRI Use of grant proposal information addendum: www.ukri.org/files/funding/tcs/grants-addendum-pdf/
2) UKRI Privacy Notice:www.ukri.org/privacy-notice/
3) UKRI Grant Terms and Conditions web page: www.ukri.org/funding/information-for-award-holders/grant-terms-and-conditions/
4) State Aid: Including but not limited to Articles 107 to 109 of the Treaty on the Functioning of the European Union, the General Block Exemption Regulation and any Enabling Regulation, as amended from time to time
5) UKRI International Due Diligence Guidance: www.ukri.org/files/funding/due-diligence-guidance-for-ukros-pdf/
6) Concordat for Engaging the Public with Research: www.ukri.org/public-engagement/research-council-partners-and-public-engagement-with-research/embedding-public-engagement/
7) UK Policy Framework for Health and Social Care Research
8) Policy and Guidelines on Governance of Good Research Conduct: www.ukri.org/about-us/policies-and-standards/research-integrity/
9) Concordat to Support Research Integrity (2012)
10) Animals (Scientific Procedures) Act 1986
11) Responsibility in the use of animals in bioscience research guidance: https://www.nc3rs.org.uk/responsibility-use-animals-bioscience-research
12) UKRI Policies and Principles for Equality, Diversity and Inclusion: www.ukri.org/about-us/policies-and-standards/equality-diversity-and-inclusion/
13) Equality Act 2010
14) Modern Slavery Act 2015
15) Advisory, Conciliation and Arbitration Service (ACAS) 'Bullying and Harassment in the Workplace: A Guide for Managers and Employers'
16) UKRI FEC Grant Guidance: https://www.ukri.org/funding/information-for-award-holders/grant-terms-and-conditions/

17) Research Outcome Reporting Requirements: www.ukri.org/funding/information-for-award-holders/research-outcomes1/help-and-guidance/
18) Research Integrity: www.ukri.org/about-us/policies-and-standards/research-integrity/
19) Concordat to Support the Career Development of Researchers
20) Open Access Policy: www.ukri.org/files/legacy/documents/rcukopenaccesspolicy-pdf/

MRC Additional Terms and Conditions

The MRC additional terms and conditions of funding supplement those of UKRI. These conditions set out operational, legislative and ethical requirements relating to medical research. The MRC reserves the right to vary these additional terms and conditions.

Research organisations and award holders have absolute responsibility for ensuring all required licenses, approvals, permissions and consent are in place before any research is undertaken and that these are followed.

Award Holders are all MRC Grant Holders and recipients of MRC Unit and Institute funding (programme leaders).

MRC reserves the right to audit at any time without prior notice:

-That required licenses, approvals, permissions and consent are in place, or were in place when the activity occurred.

-Compliance with the terms and conditions set out here.

AC1 Responsibilities of the Research Organisation: Clinicians

The research organisation is responsible for ensuring all clinicians supported by MRC funding are aware they are individually responsible for maintaining appropriate professional indemnity insurance. This should be with a professional defence organisation for any activities not covered by NHS indemnity arrangements or by additional provision made by the research organisation. MRC will not meet the costs of such cover.

The research organisation is responsible for ensuring any honorary clinical contracts required by clinical staff have been obtained prior to the start of the research.

The MRC expects the research organisations to abide by the 'UK clinical academic training in medicine and dentistry: principles and obligations' (mrc.ukri.org/documents/pdf/clinical-principles-and-obligations-report/).

AC2 Clinical Responsibilities

Clinical Fellowship holders (Clinical Research Training Fellowships, Clinician Scientist Awards or Senior Clinical Fellowships) may not work more than the time commitment for clinical duties stated in their proposal. For the majority, this will equate to up to 20% (on average over the lifetime of the grant) of their normal working hours, which they may choose to spend on NHS clinical sessions, teaching and demonstrating, or research activities beyond the scope of their fellowship. Exceptions are made for surgeons and fellows undertaking patient-oriented research, who may undertake up to 40% of their time on these duties. This is not in addition to the six hours per week all research staff supported full-time by an MRC grant or fellowship may undertake under RGC 8 of the Research Council Terms and Conditions of Research Council fEC Grants (www.ukri.org/funding/information-for-award-holders/grant-terms-and-conditions/).

AC3 Publicity for MRC-Funded Research

All research results and achievements should be communicated to the MRC Press Office (press.office@mrc.ukri.org) before publication.

Award holders must inform the MRC Press Office as soon as a paper presenting MRC-funded research is accepted for publication. The MRC reserves the right to lead on publicity when the MRC is the majority funder. The MRC Press Office must be notified at least 5 working days in advance of any publicity arising from MRC funding, and any press releases referencing the MRC must be approved by the MRC Press Office before it is released to the media.

AC4 Use of Animals

The MRC supports the principles of the 3Rs (Replacement, Reduction and Refinement). Research organisations and award holders are expected to abide by the core principles set out in the cross-funder guidance 'Responsibility in the use of animals in bioscience research: Expectations of the major research councils and charitable funding bodies' (available at www.nc3rs.org.uk) and RGC 2.2 of the Research Council Terms and Conditions (www.ukri.org/funding/information-for-award-holders/grant-terms-and-conditions/).

The provisions of the Animals (Scientific Procedures) Act 1986 must be observed. All MRC awards are made on the absolute condition that no work which is controlled by the act will begin until the necessary licences have been obtained from the Home

Office. Any recommendations arising from the MRC peer review process with regards to animal use must be followed.

When animals are purchased from commercial suppliers, UK suppliers should be used wherever possible, to minimise the risk of suffering during transport.

All research involving non-human primates must comply with the NC3Rs Guidelines: Primate accommodation, care and use (available at www.nc3rs.org.uk).

Researchers should ensure that they report animal-based studies in accordance with the ARRIVE guidelines (www.nc3rs.org.uk/ARRIVE) as far as possible, taking into account the specific editorial policies of the journal concerned.

Any new procedure likely to replace the use of animals in research or testing, reduce the numbers used or refine animal use must be reported to the MRC and disseminated through the usual channels to all those who might make use of it.

MRC is a public body legally obliged to provide information on its work to parliament and to the public, and is committed to improving transparency in public communications on animal use. MRC will make public information about the animal experiments it funds when needed (for example as anonymous examples, or in response to direct queries). MRC will resist all requests for information that might lead to the identification of places or individuals, except with the express permission of the individuals concerned.

AC5 Mouse Strains

MRC supports a central repository of mouse strains - the MRC mouse Frozen Embryo and Sperm Archive (FESA) at the Mammalian Genetics Unit, Harwell. Award holders are expected to contact FESA to highlight mouse strains engineered, or characterised using MRC funds, and are encouraged to deposit these strains with the archive.

Depositors retain ownership of strains and there is currently no charge for depositing strains to make them freely available to the academic community.

FESA aims to ensure that valuable mouse strains are safeguarded, that the need to maintain colonies of live mice for long periods of time is reduced, and that the significant investment in engineering strains is capitalised upon fully. MRC award holders planning mouse research should contact FESA at the earliest opportunity.

For help with the requirements of AC6-AC13 please contact MRC Regulatory Support Centre: mrc.ukri.org/research/facilities-and-resources-for-researchers/regulatory-support-centre/

AC6 Health Departments' Research Governance Frameworks

Research involving NHS patients, their organs, tissues or data which falls within the scope of the UK Health Departments' Research Governance Frameworks (RGF, www.hra.nhs.uk/resources/research-legislation-and-governance/research-governance-frameworks/) must comply with MRC policy on the health departments research governance frameworks (mrc.ukri.org/research/policies-and-guidance-for-researchers/clinical-research-governance/health-departments-research-governance/).

MRC requires research organisations to ensure sponsorship responsibilities are clearly identified, the research undertaken complies with the requirements of the employing organisation set out in the RGF, and that agreements and systems are in place with NHS Trusts and other partner organisations, including commercial organisations, to comply with the RGF. Systematic documentation of key decisions and approvals, particularly in relation to work with patients, their organs, tissues and data is crucial.

AC7 Human Participants in Research

MRC expects all research involving human participants to be undertaken in accordance with its policies and guidance available from mrc.ukri.org/research/policies-and-guidance-for-researchers/#ethics.These include:

-Good Research Practice (2012);
-Medical research involving adults who cannot consent (2007);
-Medical Research Involving Children (2004);
-Human Tissue and Biological Samples for Use in Research (2014);
-Personal Information in Medical Research (2000)

Research organisations and award holders have absolute responsibility for ensuring that investigations being undertaken within NHS premises, nursing or residential homes or NHS service establishments, schools, or any other organisations, do not take place without the explicit approval of the appropriate authority in advance.

Payments to healthy volunteers participating in clinical research are allowable, provided that the payment is for expense, time and inconvenience and is not at a level which would induce people to take part in studies against their better judgement. Further guidance on payments and incentives in research can be found at www.hra.nhs.uk/documents/2014/05/hra-guidance-payments-incentives-research-v1-0-final-2014-05-21.pdf

Independent Research Ethics Committee approval is required for research that involves human participants (whether patients or healthy volunteers) or records. In the case of research involving NHS patients, premises or records, this will be a NHS Research Ethics Committee (REC). Such approval is also required for certain studies of human tissues. Further guidance on when NHS

REC approval is required can be found at www.hra-decisiontools.org.uk/ethics/

In England and Wales research involving individual patient data, where the patient's consent will not be obtained, is covered by "Section 251" of The National Health Service Act 2006, and requires additional approval via the Health Research Authority's Confidentiality Advisory Group (www.hra.nhs.uk/about-the-hra/our-committees/section-251/). In Scotland, decisions on disclosure of identifiable patient information are made by Caldicott Guardians (see www.informationgovernance.scot.nhs.uk/ for further details).

In the case of social science research, the MRC recommends that award holders follow the ESRC Framework for Research Ethics (revised 2015, esrc.ukri.org/files/funding/guidance-for-applicants/esrc-framework-for-research-ethics-2015/) which highlights the responsibility of the research organisation for ensuring that the research is subject to appropriate ethics review. In some cases this review is required by an NHS REC, for further guidance please see www.hra.nhs.uk/research-community/

MRC requires the award holder to notify MRC if amendments required by a regulator or a REC will substantially affect the research question, methodology or cost previously approved.

Any serious incident arising in the course of research that has been approved by a REC should be reported immediately to the MRC, as well as to the REC. The research must be suspended until the REC has decided whether it may be continued or should be abandoned.

Research involving human participants in developing societies presents specific ethical challenges and the MRC guidelines Research Involving Human Participants in Developing Societies (mrc.ukri.org/publications/browse/research-involving-human-participants-in-developing-societies/) must be followed.

AC8 Clinical Trials

When research involves MRC-funded clinical trials, award holders must act in accordance with MRC policy on UK clinical trials regulations (mrc.ukri.org/research/policies-and-guidance-for-researchers/clinical-research-governance/clinical-trials-regulations/) in relation to ethical, sponsorship, reporting, monitoring and publication requirements.

-An independent Trial Steering Committee and Data Monitoring and Ethics Committee must be set up to oversee the conduct of the trial, with an MRC representative acting as an observer.
-MRC-funded trials must be registered with an International Standardised Randomised Control Trial Number (ISRCTN) on the ISRCTN Registry (www.isrctn.com). The unique identification number must be used in publications and provided to MRC by adding it to Researchfish within a year of the trial starting. Failure to provide this number will result in suspension of funding.
-Results of MRC-funded trials (whether positive or negative) must be published without unreasonable delay following the conclusion of the study (generally within a year of completion). Results should be reported in accordance with the recommendations in the CONSORT statement (www.consort-statement.org/). Before results are published they must be discussed by the Trial Steering Committee.
-Any contribution to an MRC-funded trial by another body, such as a pharmaceutical company (donation of drugs etc.), must be the subject of a collaboration agreement between the parties (see AC20).

AC9 Data Sharing

Award holders must comply with the MRC policy on research data sharing (mrc.ukri.org/documents/pdf/mrc-data-sharing-policy/) along with the MRC policy on sharing of research data from population and patient studies (mrc.ukri.org/publications/browse/mrc-policy-and-guidance-on-sharing-of-research-data-from-population-and-patient-studies/).

AC10 Human Fertilisation

When research involves the use of human gametes, embryos or human admixed embryos researchers must act in accordance with the Human Fertilisation and Embryology Act 1990 as amended in 2008 and 2015 (the Human Fertilisation and Embryology (Mitochondrial Donation) Regulations). This includes obtaining a research licence to undertake activities covered by the Act. Further information can be obtained from www.hfea.gov.uk/

AC11 Medical Records

When research involves the use of medical records, the award holder must act in accordance with the principles set out in the Data Protection Act 1998 and the NHS requirements to protect patient confidentiality. Advice on these requirements is available from the MRC Regulatory Support Centre.

All researchers handling personal data must have clearly established obligations to maintain confidentiality (eg formalised within policy written by their research organisations or through professional codes of conduct).

All NHS bodies should routinely inform patients that medical information may be used in research statistics, etc., and should give patients who wish to discuss any concerns an opportunity to do this (Section 251 of NHS Act 2006). Identifiable data should not be used in research if a patient has made clear that they do not wish it to be.

AC12 Removal, Use or Storage of Human Tissue

Award holders whose research involves the removal, use or storage of human tissue as specified in the relevant legislation must:

-comply with the appropriate legislation, ie the Human Tissue Act 2004 and/or the Human Tissue (Scotland) Act 2006;
-follow the relevant standards and Codes of Practice issued by the Human Tissue Authority (HTA) (the MRC Regulatory Support Centre (mrc.ukri.org/research/facilities-and-resources-for-researchers/regulatory-support-centre/) has summarised these);
-follow the MRC guidance detailed in Human Tissue and Biological Samples for Use in medical Research (2014, mrc.ukri.org/publications/browse/human-tissue-and-biological-samples-for-use-in-research/).

Where research involves the use of human tissues and cells to treat patients (human application), award holders must also:

-comply with the Human Tissue (Quality and Safety for Human Application) Regulations 2007;
-work within the applicable regulations and standards as dictated by the Human Tissue Authority, Medicines and Healthcare products Regulatory Agency (MHRA), Human Fertilisation and Embryology Authority and Health Research Authority. The UK Stem Cell Tool Kit (www.sc-toolkit.ac.uk/home.cfm) gives guidance on applicable regulatory routes, and the MHRA Innovation Office (www.gov.uk/government/groups/mhra-innovation-office) provides a regulatory advice service for regenerative medicine.

When research involves the use of human fetal tissue, or non-fetal products of conception (ie amniotic fluids, umbilical cord, placenta or membranes), researchers should follow the guidance set out in relevant Codes of Practice issued by the HTA (in particular see paragraphs 171-175 in the Code of Practice on Consent at www.hta.gov.uk/).

When research involves procedures for the removal of human tissue at post-mortem examination, researchers must also follow guidance issued by the Health Departments and Local Health Authorities.

AC13 Stem Cells

Award holders whose research involves human stem cell lines (both embryonic and adult) must:

-Abide by the UK Code of Practice for the use of Human Stem Cell lines (mrc.ukri.org/documents/pdf/code-of-practice-for-the-use-of-human-stem-cell-lines/)

-Ensure that they hold all relevant licenses, accreditations and approvals from, and abide by the Codes of Practice issued by, but not limited to, the Human Fertilisation and Embryology Authority (HFEA; see AC10), the Human Tissue Authority (HTA; see AC12), the Health Research Authority (HRA; for research ethics, gene therapy and confidentiality; see AC6, AC7, AC8), the Medicines and Healthcare products Regulatory Agency (MHRA; see AC6, AC7, AC8), the EU Tissue and Cells Directive (where applicable).

In the case of research involving human embryonic stem cells:

-Deposit a sample of every human embryonic stem cell line derived with MRC funding in the UK Stem Cell Bank; applications to deposit or access banked stem cell lines must be approved by the Steering Committee for the UK Stem Cell Bank and for the Use of Stem Cell Lines (mrc.ukri.org/research/policies-and-guidance-for-researchers/uk-stem-cell-bank-steering-committee/).

-Not pass samples of human embryonic stem cell lines to third parties other than those approved by the Steering Committee for the UK Stem Cell Bank and for the Use of Stem Cell Lines and/or the HFEA.

-Not take human embryonic stem cell lines out of the UK unless approved by the Steering Committee for the UK Stem Cell Bank and for the Use of Stem Cell Lines and/or the HFEA.

-Scientists from overseas wishing to conduct human embryonic stem cell research in the UK as visiting workers must provide a written statement from their home institution, outlining that as the employer of the visiting worker they take on the responsibilities of ensuring their employee works to and complies with the requirements of the UK Governance landscape, set out in the UK Code of Practice.

-Send copies of publications to the UK Stem Cell Bank, and agree that the UK Stem Cell Bank may post summaries of published results on their web site.

-Assist the MRC and the UK Stem Cell Bank, on request, with public engagement activities.

AC14 Use of Radioactive Substances and Neutron Irradiation in Humans

When research requires the administration of radioactive medicinal products (including in vivo neutron activation analysis in humans), researchers must follow the guidance issued by the Administration of Radioactive Substances Advisory Committee (ARSAC, www.gov.uk/government/organisations/administration-of-radioactive-substances-advisory-committee/about) and seek the relevant approval(s) as appropriate.

AC15 Genetic Modification

In accordance with the Genetically Modified Organisms (Contained Use) Regulations 2014, research organisations and individuals undertaking genetic modification must be registered with the Health and Safety Executive (HSE), undertake risk assessment and seek consent where appropriate.

Researchers who carry out genetic modification should be familiar with the legislative requirements and with the Scientific Advisory Committee on Genetic Modification (Contained Use) guidance. Advice can be obtained from HSE Head Office or from your nearest HSE Office and Knowledge Centre (www.hse.gov.uk/contact/maps/index.htm).

AC16 Dangerous Pathogens

Research organisations accommodating projects involving the use of dangerous pathogens must comply with the safeguards recommended by the Advisory Committee on Dangerous Pathogens in their guidance 'Infection at work: controlling the risk' (www.hse.gov.uk/pubns/infection.pdf), 'Biological Agents: the principles, design and operation of containment in a level 4 facility' (www.hse.gov.uk/pubns/web09.pdf) and 'Biological agents: Managing the risks in laboratories and healthcare premises' (www.hse.gov.uk/biosafety/biologagents.pdf).

AC17 Controlled Drugs

When research requires the use of one or more of the drugs controlled under the Misuse of Drugs Act, 1971 and its subsequent amendments, researchers must hold an appropriate Home Office licence in accordance with the most up to date Regulations.

AC18 Open Access Policy - Publication Repositories

To comply with the RCUK Policy on Open Access (see RGC 22 of the Research Council Terms and Conditions) the MRC requires all publications to be deposited at the earliest opportunity, and certainly within six months of publication, in Europe PubMed Central (europepmc.org/). This applies both during and after the period of funding. The condition is subject to compliance with publishers' copyright and licensing policies. Whenever possible, the article deposited should be the published version. For more information see mrc.ukri.org/research/policies-and-guidance-for-researchers/open-access-policy/

AC19 Commercial Exploitation

The research organisation should ensure that, wherever possible, the licensing of intellectual property generated from research funded by the MRC includes provision for research use by other MRC supported scientists.

Research organisations must respond to requests from the MRC to provide assurance that appropriate systems and capabilities are in place to exploit and manage intellectual property generated from MRC-funded research.

AC20 MRC Industry Collaboration Agreement

It is a condition of MRC Industry Collaboration Agreement (MICA) awards that the PI/research organisation must provide MRC Head Office with a copy of the collaboration agreement, signed by all partners, within 3 months of the date of this letter and prior to the award start date. The agreement must be consistent with the Heads of Terms submitted with the application. The grant cannot be activated, and payments, made until this document has been submitted and approved by the MRC.

AC21 Peer Review

Peer review is an integral part of the application process and ensures research of the highest calibre is funded. MRC-funded researchers are expected to contribute to this process when invited to do so, unless they have a conflict of interest (see Reviewers Handbook, mrc.ukri.org/documents/pdf/reviewers-handbook/), or where the research proposed is outside their expertise. We would typically expect an MRC-funded researcher to provide at least three reviews per year.
